# Sensorimotor transformation elicits systematic patterns of activity along the dorsoventral extent of the superior colliculus in the macaque monkey

**DOI:** 10.1038/s42003-019-0527-y

**Published:** 2019-08-02

**Authors:** Corentin Massot, Uday K. Jagadisan, Neeraj J. Gandhi

**Affiliations:** 1 Department of Bioengineering, University of Pittsburgh, Pittsburgh, PA 15260 USA; 2 Center for Neural Basis of Cognition, University of Pittsburgh, Pittsburgh, PA 15260 USA; 3 Department of Neuroscience, University of Pittsburgh, Pittsburgh, PA 15260 USA

**Keywords:** Neural circuits, Sensorimotor processing

## Abstract

The superior colliculus (SC) is an excellent substrate to study sensorimotor transformations. To date, the spatial and temporal properties of population activity along its dorsoventral axis have been inferred from single electrode studies. Here, we recorded SC population activity in non-human primates using a linear multi-contact array during delayed saccade tasks. We show that during the visual epoch, information appeared first in dorsal layers and systematically later in ventral layers. During the delay period, the laminar organization of low-spiking rate activity matched that of the visual epoch. During the pre-saccadic epoch, spiking activity emerged first in a more ventral layer, ~ 100 ms before saccade onset. This buildup of activity appeared later on nearby neurons situated both dorsally and ventrally, culminating in a synchronous burst across the dorsoventral axis, ~ 28 ms before saccade onset. Collectively, these results reveal a principled spatiotemporal organization of SC population activity underlying sensorimotor transformation for the control of gaze.

## Abstract (web summary)

Corentin Massot et al. use a linear multi-contact array to analyze neuronal activity along the superior colliculus of macaque monkeys during a delayed saccade task. They show that activity of superior colliculus neurons during the visual and pre-saccadic epochs follows a systematic spatiotemporal organization.

## Introduction

Our interactions with the environment are mediated via brain networks that transform sensory signals to motor actions at the appropriate time. In the context of gaze control, this sensorimotor transformation entails processing of incoming visual information and generating a movement command to appropriately redirect the line of sight. The superior colliculus (SC) in the midbrain modulates its activity in response to both stimulus presentation and movement generation, as well as during the interval between the two events. Like the cortex, the SC is composed of distinct layers. Its superficial layers are predominantly driven by visual processing structures like the retina and primary visual cortex, while its deeper layers communicate a broad spectrum of information with many cortical and noncortical areas^1–3^. It also has a canonical organization with established microcircuits for communication both within and across layers^4^. Finally, it has a topographic representation of visual space and for generation of gaze shifts to those locations^5^. Thus, the SC is ideally suited to study the neural correlates of sensorimotor transformation.

Despite a wealth of knowledge about the anatomical organization of the SC and the functional properties of individual SC neurons, current understanding about the link between structural and functional organization at the population level is incomplete. For instance, it is unclear whether the properties of individual neurons exhibit systematic spatiotemporal organization during the sensorimotor transformation, and how such organization is linked to the microarchitecture of the SC network. Bridging structure with function helps to not only understand the computations underlying the transformation but also build biologically inspired network models of sensorimotor learning and behavior^6,7^.

**Fig. 1:**
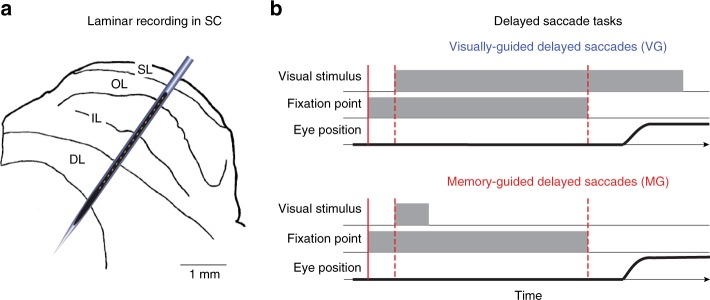
Laminar recording across the dorsoventral extent of superior colliculus (SC). **a** Schematic of SC laminar structure and laminar probe. The probe (150 µm inter-contact distance; ~300 µm diameter) is drawn roughly to scale with the SC sketch. SL (superficial layers), OL (optic layers), IL (intermediate layers), DL (deep layers) (modified from ref. ^64^). **b** Visual illustrations of the visually guided (VG) and memory-guided (MG) delayed saccade tasks. See text for details

Linear microelectrodes have recently been used for such structure-to-function mapping, particularly in cortical regions, since they enable the simultaneous measurement of neural activity across multiple layers. Indeed, this approach has provided insights into how sensory^8–11^ and cognitive processes like spatial attention^12,13^, working memory^14^, decision-making^15^, and episodic encoding^16^ are mediated as a function of depth, as well as about modes of communication between layers in driven and quiescent states^17,18^. In our case, sensorimotor transformations occurring within a single brain region provide a unique opportunity to study the link between structural organization, functional physiology, and behavior. Thus, we extended the use of the laminar probe to the SC in the subcortex to investigate the visual to motor transformation as a function of depth in monkeys performing delayed saccade tasks (Fig. 1). Our electrode penetrations were approximately orthogonal to SC surface and hence encountered neurons that responded vigorously for the same sensory and motor vectors. We were therefore able to test whether SC neurons exhibit fine-grained spatial and temporal organization that is particularly suited to implement the sensorimotor transformation. We used current–source density (CSD) analyses to obtain a veridical estimate of the relative probe depth in SC during any given penetration and align data from multiple sessions^13,18^. We found a strong and systematic depth-dependent organization for both intensity and timing of neural activity. These results reveal important spatiotemporal patterns of activity organization that advance our understanding of the neural network activity within SC. We present these results in the context of other studies of functional organization and discuss the potential implications of a structure-to-function mapping for sensorimotor transformations.

## Results

### Laminar organization of activity levels

**Fig. 2:**
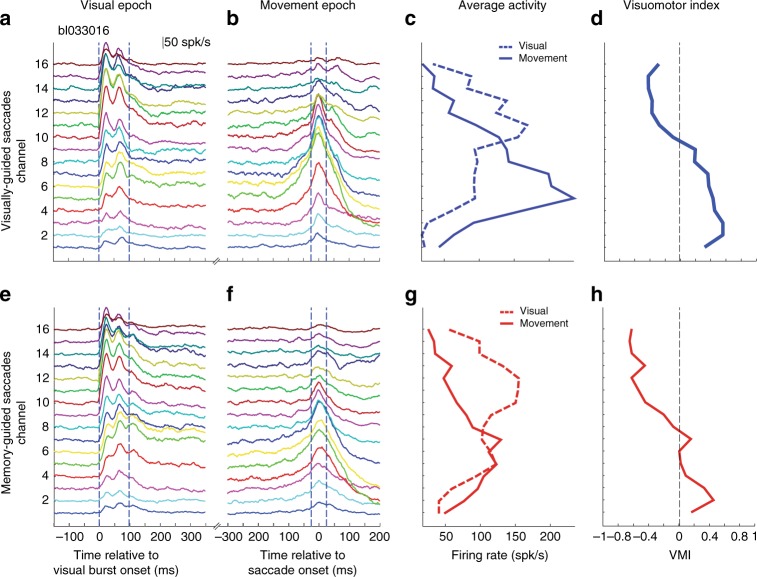
Example data of laminar recording from a single session. **a** Plots of trial-averaged spike density waveforms for each channel, aligned on visual burst onset (*t* = 0 ms). The method for aligning on burst is described in the Methods section. The waveform on each contact is scaled and shifted vertically for visualization. The dashed vertical blue lines show the boundaries of the visual epochs used in **c**. Data are from visually-guided (VG) task. **b** Trial-averaged waveforms aligned on saccade onset, using the same convention used in **a**. **c** The average firing rates during the visual (dashed blue line) and movement (solid blue line) epochs are plotted as a function of depth. **d** The visuomotor index (VMI) is plotted as a function of depth. Negative and positive VMI values denote greater visual- and movement-related activities, respectively. Vertical line denotes VMI = 0. **e**–**h** Data for memory-guided (MG) trials from the same session. Display convention is same as for **a**–**d**

Multi-unit spiking activity (MUA) and local field potentials (LFPs) were recorded on each contact of a 16-channel laminar probe that spanned the dorsoventral extent of the SC in two Rhesus monkeys performing visually guided (VG) and memory-guided (MG) delayed saccade tasks (Fig. 1). Spike density functions aligned on visual burst and saccade onsets for an individual session of VG trials are shown in Fig. 2a, b. Each trace is an average across trials for which the stimulus location matched the optimal vector estimated for the penetration (see Methods). The waveform on each contact is scaled and shifted vertically for visualization. All channels showed increased activity in response to the visual target and often exhibited two peaks. All movement bursts started before and peaked around saccade onset. Some movement bursts also displayed a second peak ~50 ms after saccade onset, which was attributed to a post-saccadic visual response. These are well-known characteristics of SC neural activity^19–21^.

A previously under-appreciated feature that emerged from the laminar probe approach is the systematic pattern of activity levels dorsoventrally along the penetration. The average firing rate in the visual epoch increased gradually with depth, reached a maximum at a relatively dorsal site (contact 11 for this example, Fig. 2c) and then decreased again for more ventral sites. The activity during the movement epoch followed the same trend except that it was shifted and peaked at a more ventral site (contact 5; contact 1 being the most ventral). We quantified the contrast between the visual and the movement-related bursts by computing a visuo-movement index (VMI) (see Methods). The index was negative (dominated by the visual response) for the most dorsal channels and gradually became positive (dominated by the movement response) for increasingly more ventral channels, before plateauing for the most ventral channels (Fig. 2d). Figure 2e–h shows these results for MG trials from the same session. The trends in spike density waveforms across depths, the average firing rates in the visual and movement epochs, and the VMI are very similar to VG trials. One notable difference, as reported previously, is that the movement burst is attenuated in MG trials^22^, particularly for the more ventral channels. This is also reflected by a slight lowering of the VMI relative to VG trials for the ventral channels.

**Fig. 3:**
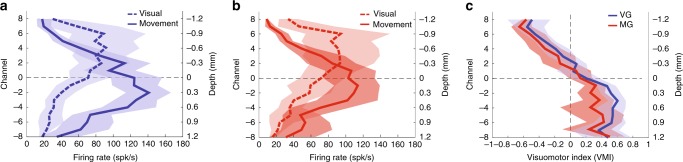
Population-averaged visual and motor activity. The peak firing rate averaged across sessions during the visual (dashed trace) and movement (solid traces) are plotted as a function of channel number or depth for visually-guided (VG) (**a**; blue traces) and memory-guided (MG) (**b**; red traces) tasks. **c** The session-averaged visuomotor index (VMI) is plotted against depth for VG (solid blue line) and MG (solid red line) trials. For all panels, the channel index is reported on the left ordinate axis. Channel 0 corresponds to the CSD reference channel that was used to align the data across sessions (see Methods). The right ordinate axis indicates the relative depth in millimeter. The blue and red translucid regions surrounding same color traces in all three panels represent 95% confidence interval of the VG and MG trials, respectively

We next sought to identify similar trends observed across sessions. We used CSD analyses to obtain a veridical estimate of the relative probe depth in SC during any given penetration and align data from multiple sessions^13,18^ (see Methods and Supplementary Figure 1). The outcomes of this CSD-guided alignment for firing rates in the visual and movement epochs are shown in Fig. 3. For VG trials (panel a), the amplitude of the visual activity plateaus from channels 2 to 6 peaks with a peak at channel 4 at 88.2 spk/s (95% confidence interval (CI) [56.1 130.3] spk/s) and then gradually decreases for the other dorsal and ventral channels. The amplitude of the movement activity peaks at channel −2 at 136.7 spk/s (95% CI [112.0 162.3] spk/s) and then gradually decreases in both dorsal and ventral directions. These general trends are similar for MG trials (panel b). The amplitude of the visual activity plateaus from channels 2 to 6 peaks with a peak at channel 6 at 92.3 spk/s (95% CI [57.8 139.2] spk/s) and then gradually decreases for the other dorsal and ventral channels. The amplitude of the movement activity peaks at channel −1 at 113.7 spk/s (95% CI [86.5 140.4] spk/s) and then gradually decreases for the other dorsal and ventral channels. The most notable difference between VG and MG trials is the smaller, but not significantly different (Wilcoxon’s rank-sum test, *P* > 0.067, for all channels), peak amplitude of movement activity for MG trials. Overall these results show that the amplitudes of two bursts are systematically organized across depths. The peak visual activity is situated between 0.75 and 1.05 mm (between channels 5 and 7) more dorsally than the peak movement activity, reflecting a visual preference for dorsal channels and movement preference for ventral channels.

We next evaluated whether the relative contributions of the visual and movement activity on each channel also follow a systematic organization along the dorsoventral axis of the SC by computing the VMI. The VMI analysis across all sessions reinforces the trends observed in the two distributions (Fig. 3c). The contrast ratio was linear from dorsal channels down to the reference channel, but then plateaued for deeper channels. It is important to note that the plateau was not due to constant firing rates of visual and movement bursts. In fact, both visual and movement activities decreased ventrally from the reference channel; however, their relative amplitudes remained constant, with the movement activity being higher. The VMI trends were similar for both VG and MG trials, as evidenced by the superposition of the CI bands during the linear part. Ventrally to the reference channel, the plateau values are slightly different (~0.5, VG trials; ~0.4, MG trials), but not statistically significantly (Wilcoxon’s rank-sum test, *P* > 0.033, for all channels). This is due to the lower firing rate of movement activity for MG trials^22^.

**Fig. 4:**
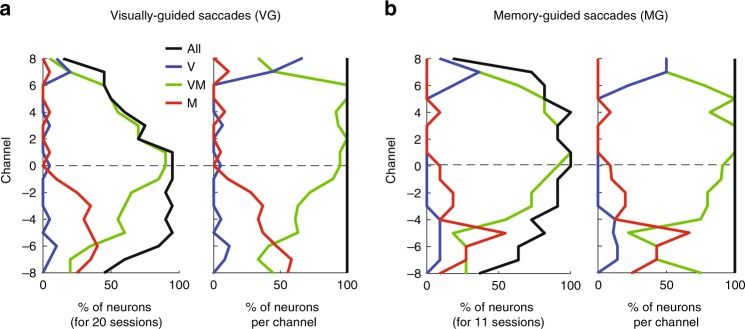
Categorization of superior colliculus (SC) neurons. Every panel shows the distributions of visual-only (V, blue trace), visuo-movement (VM, green trace), movement-only (M, red trace) and all (All, black trace) neurons as a function of channel number. Data are pooled across 20 visually-guided (VG) sessions (**a**) and 11 memory-guided (MG) sessions (**b**). In each subplot, the abscissa denotes the proportion of neurons. Left: For each channel, the neuron count (either for each category or for all neurons) across all sessions is normalized by the number of sessions (20 for VG and 11 for MG trials). Right: The neuron count is normalized individually for each channel to compensate for the non-uniform sampling of neurons across depths (i.e., the neuron count becomes 1 on every channel)

In addition to quantitative parameters like VMI, SC neurons are also parsed into visual, visuomotor (or visuo-movement), and motor (or movement) classifications (see Methods). Of the whole population of recorded channels with MUA, visuo-movement neurons constituted the majority (Supplementary Table 1). Figure 4a shows the distribution of neurons in each category as a function of depth for VG trials. The neuron count, plotted on the abscissa, is normalized to the number of sessions (left panel) and shows that more neurons were sampled in the ventral part of SC. The neuron count is also normalized individually for each channel (right panel), in order to compensate for the difference in recorded MUA across channels. Visual-only MUA were mainly found at the most dorsal channels (channels 6–8) and represented only a small proportion of the number of recorded units. This could be a consequence of the difficulty in isolating these smaller neurons^1^. Visuo-movement MUA was found across all depths and was the dominant category between channels 0 and 6. Movement-only MUA were mainly found on channels ventral to the reference channel and their proportion increased with depth. Figure 4b shows the categorization of MUA for MG trials. The distributions were qualitatively similar to VG trials. These results collectively confirm the existing view, obtained from single electrode experiments, that the activity in SC is not randomly distributed across depths, but instead follows a general principle: visual activity is predominant at dorsal depths and movement activity at ventral depths; in between, both visual and movement are mixed within the same MUA. Another crucial observation that emerges from this analysis is that SC neurons with the most vigorous movement-related burst reside in the visuo-movement, not movement, neuron category. This result has important consequence on how brainstem neurons that receive SC activity identify or decode the burst (visual or movement epoch) that triggers the movement (see Discussion).

**Fig. 5:**
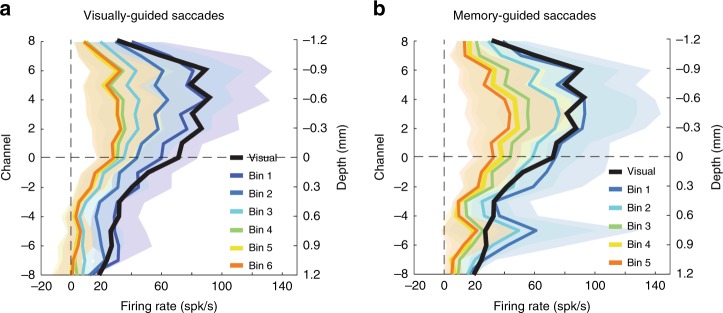
Population-averaged delay-period activity within superior colliculus (SC). Each trace represents mean activity in a specific interval as a function of depth and averaged across sessions for **a** visually-guided (VG) and **b** memory-guided (MG) tasks. The black traces is the mean activity in the visual epoch; it is identical to the dashed traces in Fig. 3a, b. The remaining traces are averages computed over non-overlapping 50 ms bins starting from the last peak of the visual burst across channels (blue, “Bin 1”) to the end of the shortest delay period (orange, “Bin 6”). The colored translucid regions surrounding the thick traces represent the 95% confidence interval computed across sessions

We next evaluated the distribution of delay-period activity as a function of depth for VG and MG trials (Fig. 5). Each color represents the across-sessions average of the baseline-corrected delay activity observed in non-overlapping 50 ms time bins, starting from the last peak of the visual burst across channels (blue, “Bin 1”) to the end of the shortest delay period (orange, “Bin 6”). The activity was, as expected, highest in the wake of the visual burst and then decreased gradually to a low-spiking rate as the delay period progresses. The strongest response stayed consistently between channels 2 and 4 throughout the delay period for both types of trials. For VG trials, the activity reached 33.0 spk/s (95% CI [14.5 61.1] spk/s) on channel 2 on Bin 6. For MG trials, the activity reached 43.6 spk/s (95% CI [13.0 83.8] spk/s) on channel 3 on Bin 5. Notably, these channels were the same that discharged maximally for the visual burst (thick black trace). For the deepest channels, we noticed a difference between the two tasks. For MG tasks, the activity was slightly higher than the peak activity of the visual burst while it remained at minimum level for VG tasks. Note that the CIs were very large and overlapping across bins, which makes this increase of activity not statistically significant in our data. Further investigation is needed to reveal the possible cause and role of this increase for MG tasks.

### Laminar organization of temporal events

**Fig. 6:**
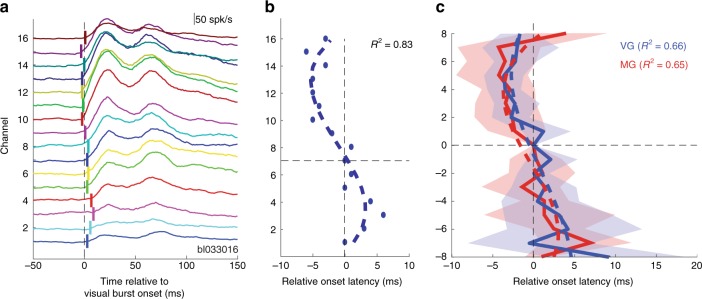
Visual latencies. **a** Data from example visually-guided (VG) session showing trial-averaged spike density functions aligned on visual burst onset (see Methods); vertical tick marks indicate the onset of the detected visual activity for each channel; corresponds to the onset of the visual activity on the visual alignment channel (here, channel 11). **b** Plot of *relative* visual burst latencies across channels for the same dataset. The data are temporally aligned on the visual onset of the reference channel (here, channel 7), which is required to averaged data across sessions. Dashed trace is a cubic fit (*r*^2^ = 0.84). **c** Session-averaged relative visual onset latencies across depths for VG (thick blue line) and MG (thick red line) trials, respectively. The dashed traces are cubic fits applied separately to the VG (blue dashed trace) and MG distributions (red dashed trace). The blue and red translucid regions surrounding the average latency estimations represent the 95% confidence interval computed across sessions; note that the CI of the reference channel is 0 because the activity of each session is temporally aligned to the latency onset of this channel

We next examined the time course of activity during the visual and the pre-saccadic epochs in order to identify potential spatiotemporal patterns along the dorsoventral axis. We first focused on the latency of the sensory response (visual burst) across depths. Data recorded during the visual epoch are typically aligned on target onset. With this type of alignment, the visual response appears 50–100 ms after target onset. However, individual neurons and local circuitry response are stochastic. A delay in this range along with the associated variability could be large enough to average out latency differences across channels. To circumvent this concern, we aligned data on the onset of the visual burst. Briefly, for each trial and for each channel, burst onset was estimated using the *Poisson surprise* detection method^23^. The channel with the most (not necessarily the earliest) detected bursts, henceforth referred to as the visual alignment channel (see Methods), was used to align data on all channels. Figure 6a shows an example session (same one as Fig. 2) with the spiking activity aligned on visual burst across; channel 11 was the visual alignment channel. Each colored, vertical mark indicates the onset of the visual activity, estimated using a two-piecewise regression-based method (see Supplementary Fig. 2 and Methods). Figure 6b plots the visual latency estimate of each channel centered on the reference channel 7, that is, the channel obtained from the CSD analysis for aligning data in depths across sessions. Note that the reference and alignment channels need not be the same. A cubic polynomial fit of the visual latencies reveals a general spatiotemporal trend for this session from dorsal to ventral depths (dashed trace, *R*^2^ = 0.83, *P* = 0.001). Figure 6c reports the session-averaged estimates of relative onset latencies in the visual epoch across depths. For VG trials, visual latencies were detected between –3.6 ms (95% CI [−6.2 −1.3] ms) on channel 5 and 9.3 ms (95% CI [2 18.8] ms) on channel −8 relative to visual burst onset on the reference channel, and the trend of longer latencies from dorsal to ventral was well captured by the cubic fit (*R*^2^ = 0.66, *P* = 0.004). For MG trials, visual latencies were detected between –4.3 ms (95% CI [−9.4 0.5] ms) on channel 5 and 7.0 ms (95% CI [1.0 12.0] ms) on channel −7 relative to visual burst onset on the reference channel, and the trend of longer latencies from dorsal to ventral was also captured by the cubic fit (*R*^2^ = 0.65, *P* = 0.004). Linear regression analysis applied to these data gives similar trends albeit a less good fit for MG trials (VG: *R*^2^ = 0.6, *P* = 0.01; MG: *R*^2^ = 0.36, *P* = 0.1). As expected, both types of tasks show the same trend in visual burst onset from dorsal to ventral depths within SC, spanning 7.3 ms (between channels 5 and −8) and 6.8 ms (between channels −5 and −7) for VG and MG tasks, respectively. This is consistent with single-synaptic transmission from one channel to another in depth, although other mechanisms are also viable (see Discussion). A similar pattern in spike timing was recently reported across cortical layers in rodents^24^.

**Fig. 7:**
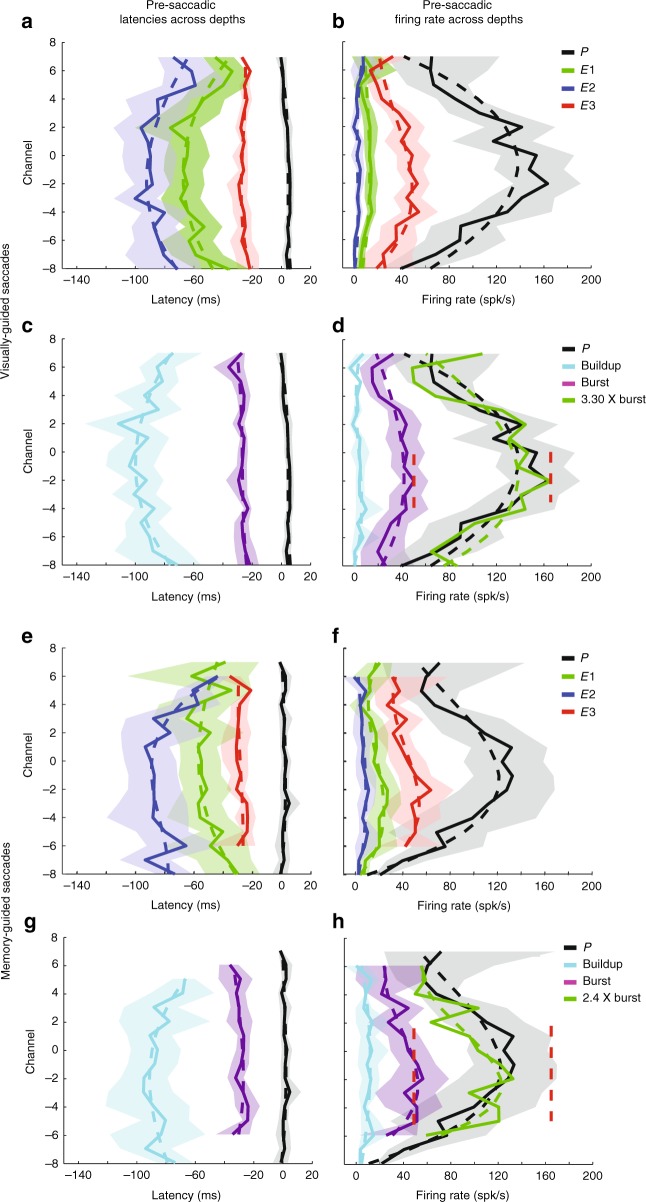
Laminar organization of onset latencies and amplitude of activity during pre-saccadic epoch. **a** Session-averaged onset latencies of events *P* (black trace), *E1* (green trace), *E2* (blue trace), and *E3* (red trace) across depths for visually-guided (VG) trials. **b** Session-averaged amplitude of the activity at the time of the events *P*, *E1*, *E2*, and *E3* across depths for VG trials. **c** Session-averaged onset latencies of peak (copy of *P* in **a**, black trace), Buildup (cyan trace), and Burst (purple trace) neural activity across depths for VG trials. **d** Session-averaged amplitude of the activity at the time of peak (copy of *P* in **a**, black trace), Buildup (cyan trace), and Burst (purple trace) onsets across depths for VG trials; a scaled version of the amplitude of Burst activity relative to peak (see Methods) is shown in green; the applied factor is 3.3. **e**–**h** Data for MG trials are shown following the same format used in **a**–**d** for VG task data. **h** Burst activity pattern was multiplied by 2.4 to match the depth-dependent pattern of peak activity. **d**, **h** Vertical dashed red lines indicate the maximum average activity of peak and Burst for VG trials. **c**, **d**, **g**, **h** Dashed lines represent a cubic fit regression. In all panels, the color-matched translucid region surrounding each trace represents the 95% confidence interval computed across sessions

Next, we analyzed the pre-saccadic epoch that leads to movement onset. Previous work has shown that SC neurons display a buildup (or prelude) and/or burst of activity during that epoch^25,26^. In these previous studies, buildup and burst activities were detected by threshold crossing of the averaged firing rate activity computed over fixed temporal window defined for each event separately. This method was designed with the only objective to detect the presence of these events. Here, we wanted to not only detect them but also reliably estimate their onset times and associated amplitudes. We developed an algorithm that, first, estimates the onset latencies of well-defined events in the trial-averaged firing rate during the pre-saccadic epoch and, second, classifies them into interpretable spiking activity such as Buildup and Burst (see Methods and Supplementary Fig. 3 for visualization). We first defined three events that can be estimated from the spiking activity: *E1*, the time of significant increase of the detrended activity compared to baseline; *E2*, the hinge point before or at *E1* and *E3*, the “hinge point” between *E2* and the time of peak activity (*P*) (see Methods and Supplementary Fig. 3g for the definition of “hinge point”^27^). The detection of each event was accompanied by a measure of reliability obtained through a bootstrapping procedure. Events that did not meet a reliability criterion were discarded. As a consequence, anywhere between zero and three events were detected for the average spiking activity of each channel. It is important to note that events *E1*, *E2*, and *E3* capture temporal characteristics of the activity and are interpretation free in terms of neural circuit mechanism. A subsequent classification procedure enabled the interpretation into Buildup or Burst activity. Supplementary Figure 4 shows the detection of events *E1*, *E2*, *E3*, and *P* for the example dataset (see Supplementary Results). Figure 7 summarizes the distribution of these events as a function of depth across all VG and MG sessions (panels a, b and e, f, respectively and see Supplementary Results for detailed explanations).

Subsequently, we used the timing of *E2* and *E3* events to classify them into Buildup and Burst categories (see Methods and Supplementary Results). Onset of Buildup was the earliest on channel 2 with an average latency of −102.7 ms (95% CI [−120.2 −83.1] ms) relative to saccade onset (Fig. 7c (cyan trace)). It occurred gradually later for increasingly more distant channels in both dorsal and ventral directions, reaching a minimum of −71.1 ms (95% CI [−97.2 −55.2] ms) relative to saccade onset. These onset estimates are similar albeit slightly later than values provided by the previous study, which analyzed the onset of Buildup 100 ms before saccade onset^25^. The difference in technique to extract onset of Buildup may explain this discrepancy. Also, our capacity to record across depths with a laminar probe allowed a finer sampling of the neural activity than this previous study that focused mainly on large neural activity. Interestingly, the onsets of Buildup activities displayed a systematic shift across depths and was well captured by a cubic fit (dashed cyan line, *R*^2^ = 0.67, *P* = 0.003) reaching a minimum on channel −1. While this analysis cannot reveal a causal relationship between the activity of neurons on different channels, the gradual change of the onset latency of Buildup may be the result of an activity that initiated in neurons situated around channel −1 and progressively later in dorsal and ventral neurons. This spatiotemporal pattern across depths may reflect a network process that leads to the generation of the movement-related burst (see Discussion). Figure 7d (cyan trace) shows that the firing rate at the onset of the Buildup activity was on average 3.4 spk/s (95% CI [−0.3 8.3] spk/s) without any significant trend across channels (cubic fit, *R*^2^ = 0.31, *P* = 0.21), indicating that the detection of the onset of Buildup corresponded to a true onset of activity relative to baseline. Figure 7c (purple trace) shows the organization of Burst onset across depths. Bursts were found at all recorded depths, from channels −8 to 7. All Bursts appeared on average −26.9 ms (95% CI [−32.0 −22.3] ms) relative to saccade onset, which is similar to a previous result (Figure 18 in ref. ^25^). All Bursts were temporally tightly aligned (i.e., synchronous, without any significant trend across depths) (cubic fit, *R*^2^ = 0.4, *P* = 0.11), regardless of whether the Burst was preceded by a Buildup. This result is indicative of a general recruitment into a “burst” mode of all neurons along the dorsoventral axis. Figure 7d (purple trace) shows that the firing rate at the onset of the Burst activity reached a maximum of 50.2 spk/s (95% CI [39.9 61.4] spk/s) on channel −2 and decreased for dorsal and ventral channels. This shift was well captured by a cubic fit (dashed purple line, *R*^2^ = 0.60, *P* = 0.01). Hence, Burst amplitude displayed a systematic shift across depths: neurons situated on channel just dorsal to the reference channel displayed the maximum firing rate, while neurons at the most dorsal and ventral positions displayed a reduced firing rate. This result implies that the Burst activity, which is related to the signal that is sent to downstream structures to control the eye movement generation, is not simply duplicated across depths but, on the contrary, its amplitude is a function of the laminar position from where it originates (see Discussion).

For MG tasks, the onset of Buildup was the earliest on channel −3 with an average latency of −95.1 ms (95% CI [−119.0 −75.5] ms) relative to saccade onset (Fig. 7g (cyan trace)). It occurred gradually later for increasing more distant channels in both dorsal and ventral directions, reaching a minimum of −66.7 ms (95% CI [−75.0 −59.9] ms) relative to saccade onset. Similar to VG trials, the onsets of Buildup activities displayed a systematic shift across depths, which was well captured by a cubic fit (dashed cyan line, *R*^2^ = 0.62, *P* = 0.02). Figure 7h (cyan trace) shows that the firing rate at the onset of the Buildup activity was on average 8.4 spk/s (95% CI [1.5 16.0] spk/s) and was not significantly different across channels (cubic fit, *R*^2^ = 0.37, *P* = 0.14). Similar to VG trials, this indicates that the detection of the onset of Buildup correspond to a true onset of activity relative to baseline. Figure 7h (purple trace) shows the organization of Burst onset across depths. Bursts were detected on nearly every neuron encountered in the penetration, from channels −6 to 6. Similar to VG trials, all Bursts appeared on average −29.1 ms (95% CI [−36.2 to −22.7] ms) relative to saccade onset and all Bursts along the dorsoventral axis were temporally tightly aligned (i.e., synchronous) (cubic fit, *R*^2^ = 0.35, *P* = 0.26). Figure 7h (purple trace) shows that the firing rate at the onset of the Burst activity reached a maximum of 55.7 spk/s (95% CI [31.4–78.2] spk/s) on channel −2 and decreased for dorsal and ventral channels. This shift was well captured by a cubic fit (dashed purple line, *R*^2^ = 0.70, *P* = 0.017). Hence, similar to VG trials, Burst amplitude displayed a systematic shift across depths.

Next, we analyzed the correspondence between the activity at Burst onset and at the peak response. To do so, we used the cubic fit computed on the distribution of the average firing rate at the onset of the Burst and at the peak *P*. A scaling factor was computed between the maximum firing rates of the two fits. Figure 7d shows the rescaled fit of the Bursts (green dashed line) for VG trials and shows the close correspondence with the fit of the peak across all depths. Hence, the peak activity of the movement epoch appears to be a scaled version of the activity at the Burst onset across depths. Figure 7h shows the same information for MG trials, and shows that the correspondence with the fit of the peak is close for ventral channels but noisier for dorsal channels. For VG and MG trials the scaling factor was 3.3 and 2.4, respectively. Note that the amplitude at the onset of Burst activity for VG and MG trials were not significantly different (Wilcoxon’s rank-sum test, *P* > 0.14). This result reveals that the lower scaling factor between Burst and the peak activity of MG trials is not the result of higher amplitude of Burst onset activity. Rather, this indicates a reduced peak activity for MG trials relative to VG trials while Burst activity reached a similar amplitude (see Discussion).

**Fig. 8:**
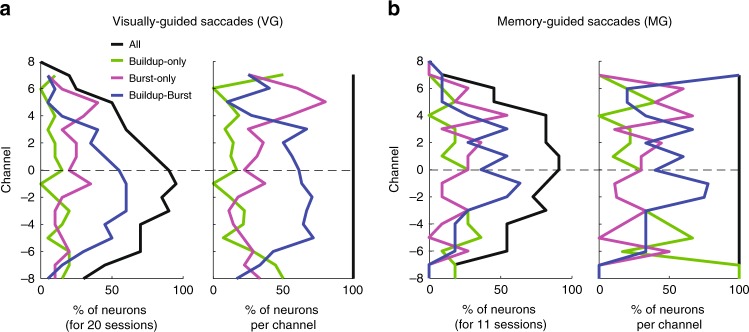
Classification of superior colliculus (SC) neurons based on pre-saccadic activity. Every panel shows the distributions of Buildup-only (green trace), Burst-only (purple trace), Buildup–Burst (blue trace) and all (black trace) neurons as a function of channel number. Data are pooled across 20 visually-guided (VG) sessions (**a**) and 11 memory-guided (MG) sessions (**b**). The figure follows the format of Fig. 4. In each subplot, the abscissa denotes the proportion of neurons. Left: For each channel, the neuron count (either for each category or for all neurons) across all sessions is normalized by the number of sessions (20 for VG and 11 for MG trials). Right: The neuron count is normalized individually for each channel to compensate for the non-uniform sampling of neurons across depths (i.e., the neuron count becomes 1 on every channel)

Finally, we looked at the classification of the spiking activity based on the type of events displayed during the pre-saccadic epoch. Similar to previous work^25^, we used three labels based on spiking activity: Buildup-only, Burst-only, and Buildup–Burst. Note that even if their exact definitions are different, buildup and burst activity by the “threshold method” of Munoz and Wurtz^25^ and Buildup and Burst events by our technique relate to similar features of the spiking activity and their detection can be used to compare the categorization of SC activity. For VG trials we found that each type of activity (Buildup-only, Burst-only and Buildup-Burst) represents 17, 30, and 53% (27, 29, and 44%, respectively, for MG trials) of the whole population of recorded activity, respectively (Supplementary Table 2). Munoz and Wurtz^25^ found that burst-only neurons were the largest proportion (6, 68 and 26%, respectively)^25^. Our results for both VG and MG trials show that Buildup–Burst activity was the largest type. The reason for this discrepancy is two-fold. First, Munoz and Wurtz used a conservative method based on threshold-crossing on the average activity measured in a window starting 100 ms before saccade onset. However, the latency distribution of Buildup onset (Fig. 7c) shows that many channels display a Buildup activity later than 100 ms before saccade onset. Hence, their method would not be able to detect these “late” Buildup onsets. Second, our method is more sensitive at detecting reliable small Buildup activity as it is based on the detection of a hinge point of the activity, which is particularly critical for the most dorsal and ventral channels where the average activity is lower. Hence, their method likely yielded a very conservative estimate of the proportion of Buildup activity. Our laminar data allow us also to plot the distribution of each type of activity across depths (Fig. 8). For VG trials, Buildup–Burst neurons were found in the more central positions between channels −6 and 4 and less at dorsal positions, Burst-only neurons are found at all depths but particularly at more dorsal positions above channel 4, and Buildup-only neurons are found at more ventral positions below channel −7. Similar distribution, albeit noisier, were found for MG trials. These results confirm the subdivision of SC intermediate layers into a dorsal subdivision that mainly contains Burst-only neurons and a ventral subdivision that mainly contains Buildup–Burst neurons^25^ (with the boundary of this subdivision situated around channel 3). Overall these results lead to a refinement of the characterization of SC neurons across depth (see Supplementary Results for additional comment on the nomenclature of SC neurons).

## Discussion

**Fig. 9:**
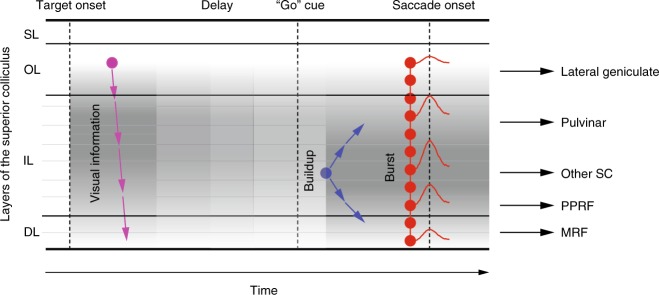
Proposed schematic representation of spatiotemporal dynamics of population activity in superior colliculus (SC). Visual information initiates (purple dot) around the superficial and optic layers (SL, OL) and systematically later in sequential order ventrally through the intermediate and deep layers (IL, DL). Neurons in the dorsal intermediate layers produced the most vigorous visual burst, shown as the darkest of the gray shade. The peak firing rate of the visual burst decreased with distance, indicated by lighter shades of gray. In the ensuing delay period, SC neurons exhibit a more sustained low-frequency activity and with a laminar organization that matches that of the visual burst. Approximately 100 ms after the “go” cue (blue dot; presumably once fixation offset is processed), neurons around the center of the intermediate layers gradually increase their firing rate. This buildup of activity appears later on adjacent layers both dorsally and ventrally (diverging blue arrows), ultimately leading to a burst synchronously across the entire dorsoventral extent of SC (red dots). Approximately 25–30 ms later, a saccade is triggered. The layers with maximal activity during the pre- and peri-saccade periods are shown in gray shades. Note that the SC layer where activity begins to accumulate after the “go” cue is also the layer that is maximally active during the burst, and that neurons maximally active during the movement phase are located more ventrally than neurons maximally active during the visual and delay periods. Rightward horizontal arrows indicate the main outputs form SC and their projection structures (adapted from ref. ^1^)

We used a multi-contact laminar probe to record simultaneously the activity of a population of neurons along the dorsoventral axis of the SC of non-human primates performing delayed saccade tasks. The categorization of the activity of each channel revealed, as proposed in Fig. 9, a visual preference for the most dorsal channels, a movement preference for the most ventral channels, and combined visual and movement responses for intermediate channels. The firing rates associated with the two events were not randomly distributed but rather changed systematically along the dorsoventral dimension (gray shades), each peaking at a certain depth and exhibiting weaker bursts with distance. Maximal activity during the visual epoch was observed ~1 mm more dorsally than during the saccade-related burst, but both were within the intermediate layers. The visuomotor index indicated a clear non-linear relationship along the dorsoventral axis from visual to motor preference. Low-frequency activity observed during the delay period was also not uniform. It followed the same spatial organization as the activity during the visual epoch: the most vigorous visual burst and the strongest delay-period activity were observed at the same depth. The onset latencies of visually-evoked activity revealed a continuous trend from dorsal to ventral channels (purple arrows). The onset latencies of both Buildup and Burst activities were detected reliably and revealed systematic spatiotemporal patterns during the pre-saccadic epoch: Buildup activity was initiated in the central part of the intermediate channels and gradually later in adjacent dorsal and ventral channels (blue arrows), while Burst activity appeared synchronously across almost all channels (red arrows). These results reveal that SC is functionally organized across depths, and its spatiotemporal patterns reflect network processes properties that were difficult to appreciate in previous studies that relied on single unit recordings. These structural patterns of SC network architecture can inform the design of biologically inspired models that implement sensorimotor transformation^6,7^.

Past efforts to correlate activity features to neuron location in SC were limited by the uncertainties of estimating the single electrode’s depth. Even the most methodical approach, for example^28^, cannot account for settling of neural tissue or of anisotropies in SC geometry (e.g., curvature). The constant inter-contact distance of the laminar probe combined with CSD alignment circumvent many limitations and thereby allow a more rigorous examination of the effects of depth. Consider, for example, the visuomotor index (VMI), a conventional ratio that contrasts the relative contributions of the visually-triggered and movement-related activities of a neuron. The relationship between VMI and depth for single electrode data (see Fig. 3b of ref. ^39^) shows a linear trend of increasing motor dominance with depth, but it is not able to reveal the saturation of VMI at deeper locations that we report here (Fig. 3c). This saturation is observed at deeper sites, where the peak activities in the visual and movement epochs begin to decrease in relatively equal amounts. These sites are ventral to the high-frequency, saccade-related burst neurons classically associated with the SC and, we speculate, that sampling bias probably contributed to their omission.

Recognizing that connectivity along the dorsoventral extent of SC is ideally suited to transform sensory signals into movement commands, previous studies have attempted to delineate the functional and anatomical substrates of the transformation. Visual latency, for example, is known to increase only modestly with depth^27^, on the order of 10–20 ms, which we confirmed (Fig. 6c). One logical prediction is that the superficial layers anatomically innervate the intermediate and deep layers of SC and that visual information is relayed through it. Indeed, in vitro slice studies in rodents have established this circuit^4^. However, this pathway is generally considered in the context of converting sensory signal into a movement command and as the primary pathway that governs short-latency express saccades^29^. While we do not dispute this hypothesis, we also cannot refute the possibility that this pathway’s central role may be to relay the visual signal to visuomotor neurons. It may even be relayed to putative motor neurons in the SC, but other inhibitory inputs may suppress its expression, and the removal of inhibition could unmask the sensory burst^30^. It is also possible that extracollicular sources may contribute to or augment the visual response relayed from the superficial layers. Indeed, visual responses of visuomotor neurons in intermediate/deep SC are delayed or absent after visual cortex lesion, while the sensory response of visual neurons in superficial layers are not as compromised^31,32^. Visual information can even be processed through the frontal eye fields, whose projections terminate in the intermediate layers of SC^33,34^. Future experiments that combine laminar probe recordings with experimental manipulations of extracollicular inputs could provide useful insights into layer-specific functional contributions.

The transient burst of the visual response was followed by low-level activity in a subset of SC neurons (Fig. 5). Given the rich balance of excitation and inhibition across all layers of the SC^35^, the persistent activity can be readily generated through network dynamics, for example^36^, although intrinsic, biophysical features likely contribute as well^37,38^. That this low-frequency activity is more prevalent in dorsal layers can be inferred from data compiled with single electrode experiments (see Fig. 7c, d of ref. ^39^), although its alignment, with rank order preserved, is best appreciated from the laminar probe data (compare Fig. 3a, b with Fig. 5). Notably, there was no transition from visually-dominant to motor-dominant layers during the delay period, which we believe has major implications on reference-frame transformation research. Previous studies have suggested that the transformation between reference frames occurs during the delay period (under appropriate task design). Correlative evidence exists for craniocentric to oculocentric representation^40,41^ and for visual, target-centered to motor, gaze-centered coordinates^42–44^. One way to reconcile these seemingly discrepant results, particularly with respect to the latter set of studies, is that the transformation may occur in dorsal, visually-dominant layers and that the population activity does not transition to the motoric layers until after the animal receives permission to produce a movement. This notion suggests that the gaze-centered signal, although thought to be a movement signal, is in fact not interpreted as a movement command, perhaps because the population activity exhibits state space dynamics that are not optimal for evoking a movement^45–47^.

Once the animal receives permission to initiate a saccade, the population activity transitions to more ventral layers (Figs. 7, 9), where neurons begin to accumulate activity ~100 ms before saccade onset. Slice experiments suggest that buildup activity is mediated by both a reduction of GABAergic inhibition^29^ and amplification by *N*-methyl-d-aspartate-mediated synaptic transmission in local excitatory circuitry within the intermediate layer neurons^48–51^. This local excitatory circuit, perhaps along with the excitatory ascending pathway^52,53^, induces buildup activity in neighboring neurons in adjacent layers at gradually longer latencies (blue arrows, Fig. 9). The continued amplification of buildup activity culminates in a synchronized burst across nearly all layers of the SC (red arrows, Fig. 9), where the peak firing rate of the movement burst appears to be a linear amplification of the cell’s activity at Burst onset^49^. Accordingly, across the active population, the neurons with the earliest Buildup onset accumulate activity the longest and therefore have the highest firing rate at both Burst onset and at peak. Given their saccade-related discharge profiles, these are the putative neurons that project to the brainstem burst generator^54,55^ and likely mediate instantaneous control of saccade velocity^56,57^. Further, the constant scaling factor between activity at burst onset to peak burst across channels (Fig. 7) provides functional evidence of linear amplification in the motor burst^49^. The amplification factor was different for VG and MG trials (~3 vs. ~2) while the activity at Burst onset was similar. Whether SC can realize this computation intrinsically remains an open question and will be the object of future research.

Finally, as suggested in Fig. 9, each laminar position within SC contains projection neurons to different structures. The laminar organization of the movement burst amplitude implies that these projection structures may decode the output of SC information in a specific way, maybe reflecting different constraints related to their role in the control of the eye movement generation. For example, the signal sent to PPRF (paramedian pontine reticular formation) may need to be temporally precise while a corollary discharge of the saccade command to pulvinar or relayed to FEF (frontal eye field) may not require such precision. Further computational modelling and multi-area recording are required to evaluate the relation between the laminar organization of the SC activity and the input signals to its projection structures.

## Methods

### Animal preparation

All experimental and surgical procedures were approved by the Institutional Animal Care and Use Committee of the University of Pittsburgh and were in compliance with the US Public Health Service policy on the humane care and use of laboratory animals. Two male, rhesus macaque monkeys (*Macaca mulatta*, ages 13 and 8 years) served as subjects. Surgical procedure details have been described previously^58^. Briefly, a recording chamber was placed on each animal to give access to the left and right colliculi. The chamber was tilted 40° posteriorly in the sagittal plane so that probe penetrations were approximately normal to the SC surface. A three-dimensional printed angular adaptor was also used to adjust the penetration angle to maximize the collinearity of the saccade vectors obtained by stimulation (see below “Neurophysiological recordings”). Head restraint was realized by fitting a thermoplastic mask individually for each animal^59^.

### Neurophysiological recordings and micrcostimulation

We used 16-channel laminar probes (Alpha-Omega, Alpharetta, USA; 150 µm inter-contact distance; ~300 µm diameter; ~1 MΩ impedance for each channel) to record neural activity across different layers of the SC (Fig. 1a). The probe was advanced with a hydraulic Microdrive (Narishige, Tokyo, Japan). When neural activity was detected on one channel, biphasic electrical stimulation (40 µA, 400 Hz, 200 ms; 200 µs pulse duration, 17 µs inter-pulse duration) was delivered to the deepest contact to determine its ability to evoke a saccade. Once verified that an intermediate layer of SC was reached, the probe was lowered further until multi-unit activity could be detected on the maximum number of contacts. Electrical stimulation was delivered on different channels to qualitatively gauge that induced saccades had similar characteristics (direction and amplitude) across depths and to estimate the average vector. This vector was used as a measure of the preferred saccade for all units recorded along the different contacts across depths. The saccade endpoint, relative to central fixation, was taken as the location of the center of the visual receptive field. The diametrically opposite location was defined as the anti-receptive field position. On a subset of sessions (*n* = 12), stimulation was applied systematically on each channel and recorded to provide a quantitative analysis (see Supplementary Results and Supplementary Fig. 6).

### Data collection

Neurophysiological signals were recorded using the Scout data acquisition system (Ripple, Salt Lake City, USA). Data recording was synchronized with the beginning of the trial, and the timing of all trial events were recorded simultaneously with raw neural activity. For each channel, the raw activity was parsed into spiking activity (high pass filter at 250 Hz and threshold at 3.5 times the root mean square) and LFP (low pass filter at 250 Hz). As the isolation of single neural activity was not achievable simultaneously on every channel, the recorded spiking activity was always considered as being from multiple units in the vicinity of each contact. A standard threshold crossing was used to determine spike times. A total of 26 sessions were recorded from 16 different grid locations. Of these, 20 were included for the analysis of the VG task. The remaining 6 were excluded because of noisy LFP signals or poor signal-to-noise ratio. In addition, 13 of the 26 sessions were also recorded with the MG task, of which 2 were excluded because of poor signal-to-noise ratio.

### Behavioral paradigms

After recovery from surgery, each animal was trained to perform standard eye-movements tasks. Eye movements were recorded using an infrared eye tracker (EyeLink 1000 from SR Research, Ottawa, Canada; RRID:SCR_009602). The camera and the infrared illuminator were situated vertically above the animal’s head. A hot mirror was tilted ~45° between the animal’s head and the display monitor. EyeLink software sampled the pupil at 1 kHz in the reflected infrared image, using its center as gaze position. Calibration of gaze position was performed for each session by having the animal fixate targets displayed at known locations on the monitor. A real-time system was used for the control of the behavioral tasks and data acquisition^60^. Animals were trained to perform two standard eye movement tasks (Fig. 1b): a VG delayed saccade task (VG) and an MG delayed saccade task. At the beginning of a VG trial, a fixation dot appeared at the center of the screen. The animal had 3000 ms to bring its line of sight towards it and maintain its fixation for 200 to 350 ms. Then, a second dot (target) appeared at a specific location on the screen. The animal had to keep its line of sight on the fixation dot for an additional 600 to 900 ms. At the end of this delay period, the fixation dot disappeared (go cue) and the animal had to make a saccade toward the target within 500 ms. The animal had to fixate the target for at least 250 ms in order to get a liquid reward and for the trial to be successful. If one of these conditions was not respected, the trial was aborted. The timeline for an MG trial was the same as for a VG trial except that the saccade target remained illuminated only for a fixed period of time (300 ms). The animal was then forced to maintain the target position in memory for the remaining of the trial. The two types of trial were typically randomly interleaved. Target locations were randomly interleaved between two locations: the center of the receptive field of the neural activity and the diametrically opposite location. We only present data from trials with the target in the response field.

### Neural activity analysis

During the delayed saccade tasks, neural activity typically increases or bursts shortly after stimulus presentation in the receptive filed and/or during a saccade directed to that target. In between the two bursts, many SC neurons exhibit a low-frequency discharge see Fig. 1 of ref. ^21^. We therefore analyzed activity separately for the visual epoch that follows target onset, the delay period that follows the visual burst and continues until fixation point is extinguished, and the movement-related epoch that ensues the go cue. Neural activity analyses were performed either on discrete spike trains or continuous spike density waveforms obtained by convolving the spikes with a kernel simulating an excitatory post-synaptic potential (growth and decay times constants of 1 and 20 ms, respectively)^61^. All data analyses were performed in MATLAB (Mathworks, Natick, USA; RRID: SCR_001622).

*Visual activity*: The study of neural response to a stimulus is traditionally performed by aligning the data on target presentation. This approach effectively ignores the trial-to-trial variability in both the actual time of target presentation (due to frame rate limit) and neuronal stochasticity. We were concerned that this variance may be greater than the time spanned for the visual response to emerge across layers. Therefore, we opted to analyze activity during the visual epoch by aligning the data on visual burst onset. We applied on the spike train for each trial and channel the “Poisson surprise” method^23^ to detect burst onset in the epoch [30 150] ms following target onset. Burst detection criteria were set to a minimum of three spikes and a surprise index of −log(0.025). For each session, the channel with the maximum number of trials with detected bursts was selected as the *alignment channel*. Trials for which no burst was detected on the alignment channel were discarded. For every remaining trial, the visual burst on the alignment channel was used to align the activity of all the other channels. Note that the activity on all the other channels was used regardless of whether a burst was detected on them. The realigned spike train for each channel was converted to a spike density function and then averaged across trials.

We next determined the relative burst onset times across the channels (Supplementary Fig. 2). First, each trial-averaged spike density waveform was baseline corrected by the mean activity computed on the same channel in the [−150 −50] ms epoch relative to visual burst onset. Next, the time of peak visual activity (Pv) was detected in the epoch [−50 150] ms around the visual burst onset. We then performed a procedure to statistically compare the distribution of activity in two sliding 20-ms windows *W1* and *W2* (Supplementary Fig. 2a). The number of points in each distribution was equal to the number of time points in the window, and *W2* was always shifted 10 ms earlier in time relative to *W1*. Initially, *W1* corresponded to the window [Pv 20 Pv] ms, and *W2* to the window [Pv−30 Pv−10] ms. Both windows slid to earlier times in 1 ms steps until *W2* reached the window [−50 −30] ms (i.e., the beginning of the visual epoch). For each instantiation, the statistical difference between the distributions of activity in *W1* and *W2* was measured using a *t* test (*P* < 0.01). Because both *W1* and *W2* started around the peak activity, their distributions were significantly different initially. Sliding to earlier times, the first instance (i.e., the beginning of *W1*) when their distributions became not significantly different and stayed not significant for the next 10 iterations defined Bv, the first time point when the activity was not significantly different from baseline (Supplementary Fig. 2b). This method was designed to account for the presence of a secondary peak in the visual epoch. Finally, a two-piecewise linear regression was computed to estimate the time point (Lv) when the spike density waveform started increasing towards Pv (Supplementary Fig. 2c, d). The regression analysis identified the intervals [Bv Lv] and [Lv Pv], respectively, where Lv minimized the sum of the residuals of the two linear fits and represented our estimate of the visual latency.

*Delay-period activity*: To examine the distribution of delay-period activity across the dorsoventral axis, we plotted average, baseline-corrected activity in 50 ms non-overlapping bins on each channel. The bins spanned from the time of the last peak of the visual burst (there were often two) to the end of the shortest delay period. The method typically yielded 5 to 6 bins for analysis. Baseline activity was the same used for visual epoch analysis.

*Movement-related activity*: Trial-averaged spike density waveforms were aligned on saccade onset, which was detected using a velocity criterion (30°/s) applied after “go” cue. Each signal was also corrected for baseline activity, defined as the average activity in the epoch [−100 0] ms relative to “go” cue. The movement-related activity was separated into two periods. The peri-saccadic or movement-related burst was quantified as the baseline-corrected average activity in the epoch [−25 25] ms centered on saccade onset. This parameter was used for analyses that computed activity levels during the burst and the visuomotor index (see below). We also defined a pre-saccadic epoch that started at the go cue and continued until saccade onset. Neural activity in this window was analyzed to detect the presence and the onset of Buildup and Burst in activity (Supplementary Fig. 3). In terms of stochastic accumulator framework^62^, this is equivalent to detecting when the activity begins to accumulate, while accounting for trends induced by baseline activity, and when it transitions into a burst. The objective was to detect up to three events in this period: *E1*, corresponding to the time of significant change of activity compared to baseline (which may include a linear trend, see below); *E2*, denoting when activity begins to accumulate and corresponding to a “hinge” point prior to *E1*; and *E3*, marking when the activity starts to burst and corresponding to a “hinge” point occurring between *E2* and *P*, the time of peak activity around saccade onset. To be able to flexibly detect one and/or two separate events (and subsequently onsets of Buildup and/or Burst activity (see below)), the Poisson surprise method used before for the estimation of the visual onset latency was discarded in favor of a two-piecewise linear regression-based approach. We sought to limit the number of ad hoc parameters while relying on statistical measures of significance through data bootstrapping. Also, this analysis was limited to saccades produced in the standard latency range of 200–400 ms.

As an initial step in our analysis, we applied a detrending procedure to remove any potential bias contributed by the low-frequency discharge from the delay period, well before the buildup and/or burst processes are engaged. A linear trend was estimated between in the epoch [300 200] ms before saccade onset. The obtained linear trend was extrapolated to the remaining time points and subtracted from the trial-averaged activity. Note that this step was only temporary and that all event detections after event *E1b* (see below) were performed on the raw, non-detrended data.

Event *E1* was defined as the first time point starting 200 ms before saccade onset for which the activity became and remained significantly different from baseline for at least 100 ms. Baseline was taken as the distribution of activity during the 100 ms period preceding the “go” cue on each trial. The number of points in the baseline distribution was equal to the number of time points in the window multiplied by the number of trials. Statistically significant difference was measured using a *t* test between the distribution of baseline activity and the distribution of activity across trials at each 1 ms time bin (*P* < 0.01). To obtain a robust estimate of *E1* and to measure CIs, subsets of trials were created through bootstrapping. An estimate of *E1* (denoted *E1b*) was obtained for each bootstrap iteration. Supplementary Figure 3a provides a visualization of the method for one bootstrap. We used 100 bootstrapped estimates (Supplementary Fig. 3c) and defined *E1* as the average of all 100 *E1b* (Supplementary Fig. 3e, left). CIs were used as a measure of the reliability of the estimation of *E1* and computed as the 95% quantile of the *E1b* distribution (Supplementary Fig. 3e, right). For all events, CIs were normalized with the average size of the search window. For *E1b*, the size of the search window was constant at 200 ms. To exclude unreliable estimation of *E1*, a 0.6 threshold was applied on the total range of CIs. This threshold was the only ad hoc parameter in the algorithm and the same value was used for all events (which was allowed by the normalization of the CIs). The threshold value was chosen in order to remove very unreliable estimations (e.g., for event *E1*, the excluded estimations had CIs superior to 120 ms). Changing the threshold value (e.g., to 0.5 or 0.4) did not alter the general trend of the results. With this method, *E1* is the latest time point of statistically significant change from baseline activity. The actual change, indicating the onset of accumulation, most likely occurs prior to it. Thus, we next operated on the non-detrended averaged spike density waveform to obtain a better estimate of the actual time of change from baseline activity, while imposing the constraint of *E1*.

Event *E2* denotes the time point before *E1* when the activity starts deviating from the ongoing activity and displays what we refer to a “hinge point,” which we define as the time point at which the rate of change of the spiking activity deviates from its current trend (see Supplementary Fig. 3g for a general visualization). The hinge point was detected by finding the best piecewise linear regression for the relevant data points. For each bootstrapped estimation *E1b*, a piecewise linear regression was performed on the intervals [*E1b*–100 *Hb*] and [*Hb* *E1b*], respectively, and the value of *Hb* that minimized the sum of the residuals of the total fit was the estimate of *E2b*. The combination of the search windows of *E1b* and of *E2b* relative to *E1b* implies that *E2b* was searched in a potentially very large window starting 300 ms before saccade onset and ending as late as *P*. By definition, *E2b* always preceded *E1b* or was equal to it. *E2* was taken as the average of all the bootstrapped estimates of *E2b*. As before, we performed 100 bootstrapped estimations and computed the CIs, which were normalized by 100 ms. To exclude an unreliable estimate of *E2*, a 0.6 threshold was applied on the total range of the normalized CIs (Supplementary Fig. 3e, f).

Event *E3* marks when the activity starts to burst and corresponds to the hinge point occurring between *E2* and *P*, the time of peak activity around saccade onset. For each round of bootstrapping, we obtain *E2b* as stated above and an estimate of the time of peak activity (*Pb*). Then we estimated the hinge point *E3b* by fitting a two-piecewise linear regression between *E2b* and *Pb*. The detection of the slopes of the two linear regressions around *E3b* is stored for statistical analysis. By definition, *E3b* is always between *E2b* and *Pb*. *E3* was taken of the average of all the bootstrapped estimates *E3b*. To exclude an unreliable estimate of *E3*, a 0.6 threshold was applied on the total range of the CIs, which normalized by the average interval between *E2b* and *Pb*. *E3* was considered a hinge point only if the CIs of the distribution of the slopes of the two linear regressions before and after all *E3b* were not overlapping (this is a conservative measure of significance). Hence, all estimated *E3* values correspond to a hinge point with a significant change of rate of activity around this time (Supplementary Fig. 3d, f, g). Also, *P* was taken as the average of all *Pb* and CIs were computed (Supplementary Fig. 3c, e).

*Classification of events into Buildup and Burst activity*: Once estimated, these events were used to categorize the pre-saccadic discharge pattern into Buildup or Burst activity (Supplementary Fig. 3h). To avoid any confusion with previous literature^25,26^, we used the terms Buildup and Burst with a capital letter to refer to these events with a definition specific to this study. We started with the subset of channels across all sessions for which both *E2* and *E3* were both detected and were significantly different from each other (i.e., their confidence intervals did not overlap). A distribution of the event times exhibited visual separation around −50 ms relative to saccade onset (see Supplementary Results and Supplementary Fig. 5). We therefore used this boundary to distinguish Buildup (<−50 ms) from Burst (>−50 ms) events. We similarly examined the distribution of times when only one of the two events was detected, and we once again used the −50 ms boundary criterion to classify the event. Thus, it was possible that activity associated with event *E2* (resp. *E3*) in the absence of *E3* (resp. *E2*) to be classified as a Burst (resp. Buildup) activity.

### Neuronal activity categorization

Existing literature uses nomenclature for categorizing the cell types in the SC (and other structures, such as FEF) depending on their activity during the visual or the peri-saccadic movement epoch. We also performed analyses to determine how neurons based on this classification vary with depth. Only channels with statistically significant neural activity in at least one of the two intervals were included in the subsequent analyses. To do so, we used spike density data aligned on visual burst onset but not baseline corrected. The significance was measured using a statistical test carried out between the distribution across trials of the activity during the visual epoch and the activity during baseline (Wilcoxon’s rank-sum test, *P* < 0.001). To discard very low activity after baseline correction, an additional low threshold (10 spk/s) was used on the trial-averaged baseline-corrected activity and averaged across the visual epoch. We used the same procedure to measure the statistical significance of the activity during the movement epoch with data aligned on saccade onset and baseline activity measured on data aligned on go cue (see above). Based on this measure, the MUA on each channel was categorized as follows: visual-only activity (significant visual activity and not significant movement activity); visuo-movement activity (significant visual and movement activity); movement-only activity (not significant visual activity and significant movement activity).

The visuo-movement index (VMI) contrasts the visual and the movement activity of MUA during the delayed saccade tasks. The visual activity (*V*) is the baseline-corrected average activity in the epoch [0 100] ms following visual burst onset (see above “Neural data analyses”). The movement activity (*M*) is the baseline-corrected average activity in the peri-saccadic epoch [−25 25] ms centered on saccade onset. We defined the index as VMI = (*M* − *V*)/(*M* + *V*). With this formulation, VMI = −1 corresponds to a visual neuron with no saccade-related activity, while VMI = +1 corresponds to a movement neuron with no visual response. We also computed VMI trends when average activity in each epoch is replaced with peak activity and when baseline activity for the movement epoch is measured before target onset. VMI results were qualitatively similar for these variations.

### Depth alignment of multiple sessions

The above analyses focus on population neural activity across SC layers within a session. To assess reliability, data must be averaged across sessions, which required appropriate alignment of data collected in each penetration.

One consideration is to use microdrive readings, which corresponds to the absolute depth of the linear probe from the dura. However, it is not a reliable measure because of two main limitations. First, the daily setup of the recording equipment introduces slight configuration changes that are difficult to control for (notably the initial position of the probe). Second, the viscosity of the cerebral tissue, which can change both within and across sessions, introduces an inherent variability. This makes it unlikely that the same absolute depth of the probe, as indicated by the microdrive, corresponds to the same relative position within SC. While the effects of the recording setup can be mitigated^28^, the viscosity of the tissue cannot be controlled for. To overcome these limitations, we used an objective method based on features in the CSD analysis of the LFP signals from the visual epoch (see Supplementary Fig. 1). CSD computes the second spatial derivative of LFPs and provides an estimate of the distribution of the current sinks and sources as a function of space and time in a volume of tissue. We estimated the CSD using the *csdplotter* toolbox that contains the implementation of the iCSD method (https://github.com/espenhgn/CSDplotter)^63^. Supplementary Fig. 1 demonstrates the utility of the CSD method for the depth alignment of two datasets recorded at two different locations 1 mm apart along the same penetration. Panels a and d display average LFP signals recorded at each contact at two locations 1 mm apart along the same penetration. The LFPs showed a large decrease reflecting the input current following the display of the target. The CSD plots of the two datasets (panels b and e) revealed a strong current sink (orange bands) occurring after target onset and which encompassed almost 1 mm of SC tissue. This feature was present in the recordings at both locations but translated in depth. The lower bound of the sink pattern was at 1.28 and 1.87 mm in panels b and e, respectively. Such a strong sink appeared systematically after target onset across all recording sites (analysis subject to a future manuscript). We exploited this feature to align the data across sessions. The lower bound limit of the sink pattern was used as a reference for estimating the relative depth of the probe. Supplementary Figure 1c, f shows the average profile of the CSD in a 150 ms window starting before burst onset. The transitions from negative to positive CSD was detected automatically and visually inspected to account for the rare cases when the sink pattern was not continuous due to decreased SNR of the LFP. To assess the utility of the CSD alignment method, we compared the relationship between depth and visuomotor index (VMI, see below) at the two depths (panel g). The two VMI plots appear very similar but shifted in depth. After alignment using the CSD method, the two VMI graphs overlap very well (panel h).

For aligning data by depth across recording sessions, the channel closest to the transition from negative to positive CSD was identified. We termed it the reference channel and assigned it index 0 in plots presenting data after alignment. The indices of the remaining channels were shifted accordingly. Note that the alignment was done in terms of channel index and not in actual mm, which would require an interpolation of the signals between the channels. Given that the inter-contact distance of the probe is 150 µm, the maximum error of alignment based on channel index is 75 µm. Supplementary Figure 1i shows the average CSD profile of all sessions and for both VG and MG trials. It highlights the robustness of the detection of the CSD reference channel and the systematic presence of a sink pattern above it (i.e., negative values of the CSD profile). Note that if depth alignment between datasets is necessary (i.e., the probe’s depth position is different across datasets), then there exists at least one non-overlapping channel between them. Thus, if any of these non-overlapping channels contains MUA, then the total number of channels across which data can be analyzed may be larger than the total number of channels of the probe. In practice, only few channels were added and all data analysis that required depth alignment will be presented between channels −8 and 8 (17 channels).

The results show that the alignment is precise enough to unveil a systematic functional organization of the intermediate layers of SC. Here, we interpret the strong sink pattern occurring during the visual epoch as the input current reflecting the visual input from target presentation. Based on known SC neuroanatomy^1^, this input is most likely occurring in the optic and superficial layers. Previous works in the primary and the frontal cortex^8,12,13,17,18^ have also used CSD analysis to align data across sessions. Most of these methods use a sink/source pair pattern occurring early on during the trial and interpreted as the sensory input occurring in layer 4. One study, in addition, uses a distinctive pattern of the LFP to measure the depth of the dura while recording in SEF^18^. In the absence of such anatomical marker, methods based only on a systematic pattern in the CSD, like our SC method or like the cortical methods, can only align data relatively to an arbitrary reference. Histological verification and/or neuroanatomical experiments are required to verify the reference location. We did not pursue this option as the animals are still in use. However, the consistency in the spatial and temporal distributions of activity patterns and burst onsets, respectively, in visual and movement epochs across sessions provides confidence in the CSD alignment method for inferring laminar composition and function. As next step, it would be valuable to employ CSD profiles to more precisely identify the superficial and deeper boundaries of the SC. To this end, spikes and LFPs would need to be recorded on at least one and ideally more contacts beyond the dorsal and ventral boundaries to evaluate the CSD. Additionally, interpretations will need to account for the different neuronal morphologies observed across layers^1^ and for the consequences of mechanical damage induced by repeated electrode penetrations across the duration of the experiments.

### Statistics and reproducibility

Trial-averaged activity was computed using large numbers of trials. CIs were measured using bootstrapping (1000 bootstraps, *bootci* in MATLAB). Normality assumption was systematically assessed using a Kolomogorov–Smirnov test (*kstest* in MATLAB). When the hypothesis of normality was not rejected, a parametric test was applied (*t* test, *ttest* in MATLAB), otherwise a non-parametric test was used (Wilcoxon’s rank-sum test, *ranksum* in MATLAB). All tests were two tailed.

To measure trends of latencies and activity amplitude across depths, we fitted a cubic function to the data of the form (*ax*^3^ + *bx*^2^ + *cx* + *d*), where *x* is the depth within SC measured using channel index^15^. We chose higher order fits to capture non-monotonic trends across depths. *R*^2^ values were reported to assess the goodness of fit. A *P* value for each cubic fit was obtained using a permutation test. For each permutation, the index of the SC depth was shuffled and a new shuffled *R*^2^ value was obtained by fitting a new cubic function. A total of 1000 permutations were done. The *P* value was computed as the number of time shuffled *R*^2^ values were superior or equal to the un-shuffled *R*^2^ value, divided by the number of permutations. A *P* value inferior to 0.05 indicated a significant fit and, hence, that the data displayed a trend across depths.

### Reporting summary

Further information on research design is available in the Nature Research Reporting Summary linked to this article.

## Supplementary information

Supplementary Information

Reporting Summary

## Data Availability

All data are available upon reasonable requests to the corresponding author.

## Code availability

Custom code for general analysis of laminar recording of the Superior Colliculus and events detection is available at https://github.com/CorentinMassot-NeuroAI/LaminarSC-CodeEventsAnalysis.

## References

[1] May PJ (2006). The mammalian superior colliculus: laminar structure and connections. Prog. Brain Res..

[2] Basso MA, May PJ (2017). Circuits for action and cognition: a view from the superior colliculus. Annu. Rev. Vis. Sci..

[3] Krauzlis RJ, Lovejoy LP, Zenon A (2013). Superior colliculus and visual spatial attention. Annu. Rev. Neurosci..

[4] Isa T, Hall WC (2009). Exploring the superior colliculus in vitro. J. Neurophysiol..

[5] Gandhi NJ, Katnani HA (2011). Motor functions of the superior colliculus. Annu. Rev. Neurosci..

[6] Murakami M, Mainen ZF (2015). Preparing and selecting actions with neural populations: toward cortical circuit mechanisms. Curr. Opin. Neurobiol..

[7] Song H. Francis, Yang Guangyu R., Wang Xiao-Jing (2016). Training Excitatory-Inhibitory Recurrent Neural Networks for Cognitive Tasks: A Simple and Flexible Framework. PLOS Computational Biology.

[8] Maier A, Aura CJ, Leopold DA (2011). Infragranular sources of sustained local field potential responses in macaque primary visual cortex. J. Neurosci..

[9] Self MW, van Kerkoerle T, Super H, Roelfsema PR (2013). Distinct roles of the cortical layers of area V1 in figure-ground segregation. Curr. Biol..

[10] Kajikawa Y, Smiley JF, Schroeder CE (2017). Primary generators of visually evoked field potentials recorded in the macaque auditory cortex. J. Neurosci..

[11] Wohlgemuth MJ, Kothari NB, Moss CF (2018). Functional organization and dynamic activity in the superior colliculus of the echolocating bat, *Eptesicus fuscus*. J. Neurosci..

[12] van Kerkoerle T, Self MW, Roelfsema PR (2017). Layer-specificity in the effects of attention and working memory on activity in primary visual cortex. Nat. Commun..

[13] Nandy AS, Nassi JJ, Reynolds JH (2017). Laminar organization of attentional modulation in Macaque visual area V4. Neuron.

[14] Bastos AM, Loonis R, Kornblith S, Lundqvist M, Miller EK (2018). Laminar recordings in frontal cortex suggest distinct layers for maintenance and control of working memory. Proc. Natl. Acad. Sci. USA.

[15] Chandrasekaran C, Peixoto D, Newsome WT, Shenoy KV (2017). Laminar differences in decision-related neural activity in dorsal premotor cortex. Nat. Commun..

[16] Maass A (2014). Laminar activity in the hippocampus and entorhinal cortex related to novelty and episodic encoding. Nat. Commun..

[17] Ninomiya T, Dougherty K, Godlove DC, Schall JD, Maier A (2015). Microcircuitry of agranular frontal cortex: contrasting laminar connectivity between occipital and frontal areas. J. Neurophysiol..

[18] Godlove DC, Maier A, Woodman GF, Schall JD (2014). Microcircuitry of agranular frontal cortex: testing the generality of the canonical cortical microcircuit. J. Neurosci..

[19] Marino RA (2012). Linking visual response properties in the superior colliculus to saccade behavior. Eur. J. Neurosci..

[20] Mays LE, Sparks DL (1980). Dissociation of visual and saccade-related responses in superior colliculus neurons. J. Neurophysiol..

[21] McPeek RM, Keller EL (2002). Saccade target selection in the superior colliculus during a visual search task. J. Neurophysiol..

[22] Edelman JA, Goldberg ME (2001). Dependence of saccade-related activity in the primate superior colliculus on visual target presence. J. Neurophysiol..

[23] Hanes DP, Thompson KG, Schall JD (1995). Relationship of presaccadic activity in frontal eye field and supplementary eye field to saccade initiation in macaque: Poisson spike train analysis. Exp. Brain Res..

[24] Plomp G, Michel CM, Quairiaux C (2017). Systematic population spike delays across cortical layers within and between primary sensory areas. Sci. Rep..

[25] Munoz DP, Wurtz RH (1995). Saccade-related activity in monkey superior colliculus. I. Characteristics of burst and buildup cells. J. Neurophysiol..

[26] Glimcher PW, Sparks DL (1992). Movement selection in advance of action in the superior colliculus. Nature.

[27] Wurtz RH, Mohler CW (1976). Enhancement of visual responses in monkey striate cortex and frontal eye fields. J. Neurophysiol..

[28] Hafed ZM, Chen CY (2016). Sharper, stronger, faster upper visual field representation in primate superior colliculus. Curr. Biol..

[29] Isa T, Endo T, Saito Y (1998). The visuo-motor pathway in the local circuit of the rat superior colliculus. J. Neurosci..

[30] Jagadisan UK, Gandhi NJ (2016). Disruption of fixation reveals latent sensorimotor processes in the superior colliculus. J. Neurosci..

[31] Schiller PH, Stryker M, Cynader M, Berman N (1974). Response characteristics of single cells in the monkey superior colliculus following ablation or cooling of visual cortex. J. Neurophysiol..

[32] Takaura K, Yoshida M, Isa T (2011). Neural substrate of spatial memory in the superior colliculus after damage to the primary visual cortex. J. Neurosci..

[33] Helminski JO, Segraves MA (2003). Macaque frontal eye field input to saccade-related neurons in the superior colliculus. J. Neurophysiol..

[34] Sommer MA, Wurtz RH (2001). Frontal eye field sends delay activity related to movement, memory, and vision to the superior colliculus. J. Neurophysiol..

[35] Phongphanphanee P (2014). Distinct local circuit properties of the superficial and intermediate layers of the rodent superior colliculus. Eur. J. Neurosci..

[36] Lim S, Goldman MS (2013). Balanced cortical microcircuitry for maintaining information in working memory. Nat. Neurosci..

[37] Ghitani N, Bayguinov PO, Basso MA, Jackson MB (2016). A sodium afterdepolarization in rat superior colliculus neurons and its contribution to population activity. J. Neurophysiol..

[38] Saito Y, Isa T (1999). Electrophysiological and morphological properties of neurons in the rat superior colliculus. I. Neurons in the intermediate layer. J. Neurophysiol..

[39] Ikeda T (2015). Spatio-temporal response properties of local field potentials in the primate superior colliculus. Eur. J. Neurosci..

[40] Lee J, Groh JM (2012). Auditory signals evolve from hybrid- to eye-centered coordinates in the primate superior colliculus. J. Neurophysiol..

[41] Caruso VC, Pages DS, Sommer MA, Groh JM (2018). Beyond the labeled line: variation in visual reference frames from intraparietal cortex to frontal eye fields and the superior colliculus. J. Neurophysiol..

[42] Sadeh M, Sajad A, Wang H, Yan X, Crawford JD (2015). Spatial transformations between superior colliculus visual and motor response fields during head-unrestrained gaze shifts. Eur. J. Neurosci..

[43] DeSouza JF (2011). Intrinsic reference frames of superior colliculus visuomotor receptive fields during head-unrestrained gaze shifts. J. Neurosci..

[44] Sadeh M, Sajad A, Wang H, Yan X, Crawford JD (2018). The influence of a memory delay on spatial coding in the superior colliculus: Is visual always visual and motor always motor?. Front. Neural Circuits.

[45] Kaufman MT, Churchland MM, Ryu SI, Shenoy KV (2014). Cortical activity in the null space: permitting preparation without movement. Nat. Neurosci..

[46] Churchland MM, Yu BM, Ryu SI, Santhanam G, Shenoy KV (2006). Neural variability in premotor cortex provides a signature of motor preparation. J. Neurosci..

[47] Jagadisan, U. K. & Gandhi, N. J. Population temporal structure supplements the rate code during sensorimotor transformations. *bioRxiv*10.1101/132514 (2018). 10.1016/j.cub.2022.01.015 PMC8930729 35114097

[48] Pettit DL, Helms MC, Lee P, Augustine GJ, Hall WC (1999). Local excitatory circuits in the intermediate gray layer of the superior colliculus. J. Neurophysiol..

[49] Saito Y, Isa T (2003). Local excitatory network and NMDA receptor activation generate a synchronous and bursting command from the superior colliculus. J. Neurosci..

[50] Özen G, Augustine GJ, Hall WC (2000). Contribution of superficial layer neurons to premotor bursts in the superior colliculus. J. Neurophysiol..

[51] Phongphanphanee P, Kaneda K, Isa T (2008). Spatiotemporal profiles of field potentials in mouse superior colliculus analyzed by multichannel recording. J. Neurosci..

[52] Ghitani N (2014). Excitatory synaptic feedback from the motor layer to the sensory layers of the superior colliculus. J. Neurosci..

[53] Vokoun CR, Jackson MB, Basso MA (2010). Intralaminar and interlaminar activity within the rodent superior colliculus visualized with voltage imaging. J. Neurosci..

[54] Rodgers CK, Munoz DP, Scott SH, Pare M (2006). Discharge properties of monkey tectoreticular neurons. J. Neurophysiol..

[55] Raybourn MS, Keller EL (1977). Colliculoreticular organization in primate oculomotor system. J. Neurophysiol..

[56] Smalianchuk I, Jagadisan UK, Gandhi NJ (2018). Instantaneous midbrain control of saccade velocity. J. Neurosci..

[57] Jagadisan, U. K. & Gandhi, N. J. Removal of inhibition uncovers latent movement potential during preparation. *Elife* **6**, 10.7554/eLife.29648 (2017). 10.7554/eLife.29648 PMC5650474 28891467

[58] Katnani HA, Gandhi NJ (2011). Order of operations for decoding superior colliculus activity for saccade generation. J. Neurophysiol..

[59] Drucker CB, Carlson ML, Toda K, DeWind NK, Platt ML (2015). Non-invasive primate head restraint using thermoplastic masks. J. Neurosci. Methods.

[60] Bryant CL, Gandhi NJ (2005). Real-time data acquisition and control system for the measurement of motor and neural data. J. Neurosci. Methods.

[61] Thompson KG, Hanes DP, Bichot NP, Schall JD (1996). Perceptual and motor processing stages identified in the activity of macaque frontal eye field neurons during visual search. J. Neurophysiol..

[62] Peel TR, Dash S, Lomber SG, Corneil BD (2017). Frontal eye field inactivation diminishes superior colliculus activity, but delayed saccadic accumulation governs reaction time increases. J. Neurosci..

[63] Pettersen KH, Devor A, Ulbert I, Dale AM, Einevoll GT (2006). Current–source density estimation based on inversion of electrostatic forward solution: effects of finite extent of neuronal activity and conductivity discontinuities. J. Neurosci. Methods.

[64] Ma TP, Graybiel AM, Wurtz RH (1991). Location of saccade-related neurons in the Macaque superior colliculus. Exp. Brain Res..

