# Supplementary material for: Sensorimotor transformation elicits systematic patterns of activity along the dorsoventral extent of the superior colliculus in the macaque monkey
Source: Commun Biol. 2019 Aug 2;2:287. doi: 10.1038/s42003-019-0527-y (PMC6677725; doi:10.1038/s42003-019-0527-y)
Supplement: Supplementary file 1 — Supplementary Information [file 42003_2019_527_MOESM1_ESM.docx]

**Supplementary Figure 1**


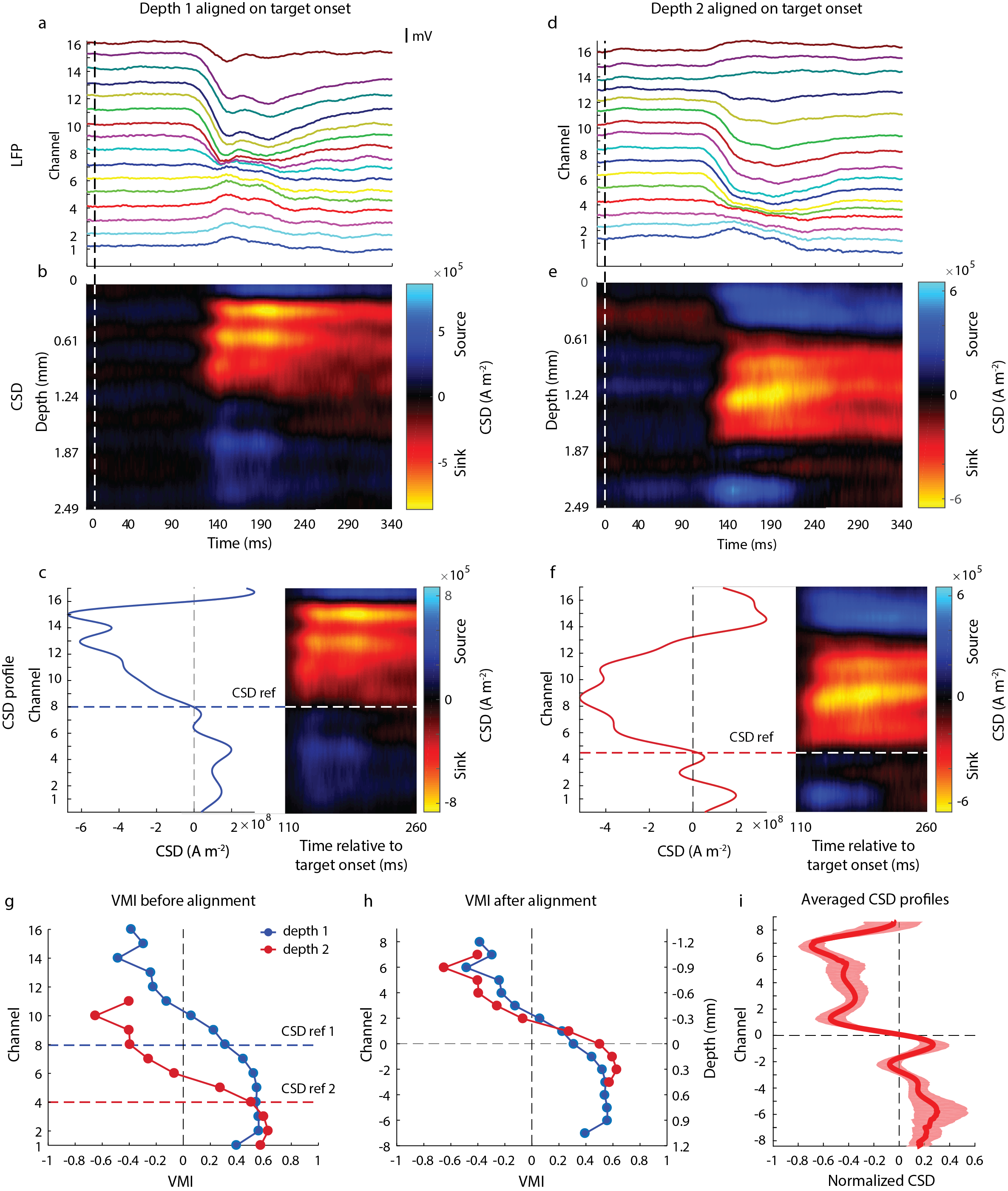


**­­­Supplementary Figure 1. Alignment procedure based on LFP and CSD profiles.** (a) Plots of trial-averaged LFP signals for one recording session with a 16-channel linear probe during a VG task; signals have been offset vertically to distinguish the activity on the different channels; channel 1 and 16 correspond to the most ventral and most dorsal contacts, respectively. Data are aligned on target onset. (b) Plot of CSD signal obtained from the LFP signals in (a), obtained with the iCSD method in the *csdplotter* toolbox (<https://github.com/espenhgn/CSDplotter>)[^1^](#_ENREF_1). Negative and positive values correspond to current sinks and sources, respectively. (c) Another representation of the CSD profile (left panel). It shows the temporal average of the CSD values computed over the 150ms window following the sink onset (right panel). The horizontal line represents the depth of the crossing from negative to positive CSD values. This crossing is taken as the depth of reference for the alignment procedure. (d-f) Plots resulting from the same analysis as (a-c) for data recorded during the same session and for the same penetration but with the probe ~1mm shallower in SC. (g) The VMI as a function of depth for the two examples presented in (a) (depth 1 in blue) and (d) (depth 2 in red); the horizontal dashed lines indicate the CSD reference channels. (h) The VMI traces of the two examples are replotted after alignment based on the CSD analysis; the channels’ index is reported on the left y axis. Channel 0 corresponds to the reference channel. The right y axis indicates the relative depth in mm. (i) The trace shows the CSD profile averaged across all sessions, not just the two examples from above, and for both VG and MG trials. The red translucid region surrounding the average CSD profile represents 95% confidence interval.

**Supplementary Figure 2**

**
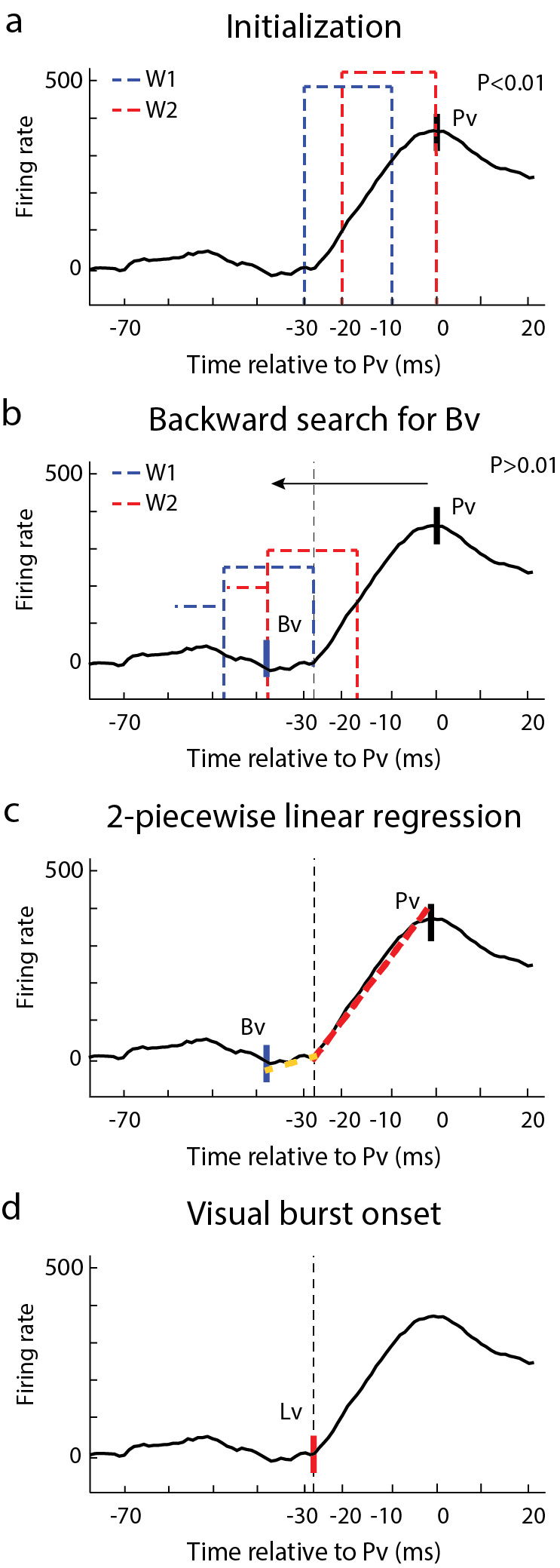
**

**Supplementary Figure 2. Visual latency detection.** (a) Baseline-corrected average spike density waveform (solid black trace). *Pv* is the peak activity detected in the [-50 150]ms epoch after target onset. The x-axis represents time relative to Pv for display purposes. The two sliding windows *W1* (dashed red) and *W2* (dashed blue) are represented at their initial positions; a statistical difference between the distributions of activity in *W1* and *W2* is measured using a t-test; the test is significative at the initialization (P<0.01, N=20 (windows’ size)). (b) Both windows slide to earlier times in 1ms steps; the t-test is performed at each time step; *Bv* is the first time point (measured relative to the beginning of *W1*) when the *t*-test indicates a non-significant difference and stays not significant for the next 10 steps. (c) A two-piecewise linear regression is computed between *Bv* and *Pv.* (d) *Lv* is the time point that minimizes the residuals of the two-piecewise linear regression and represents the onset of the visual burst.

**Supplementary Figure 3**


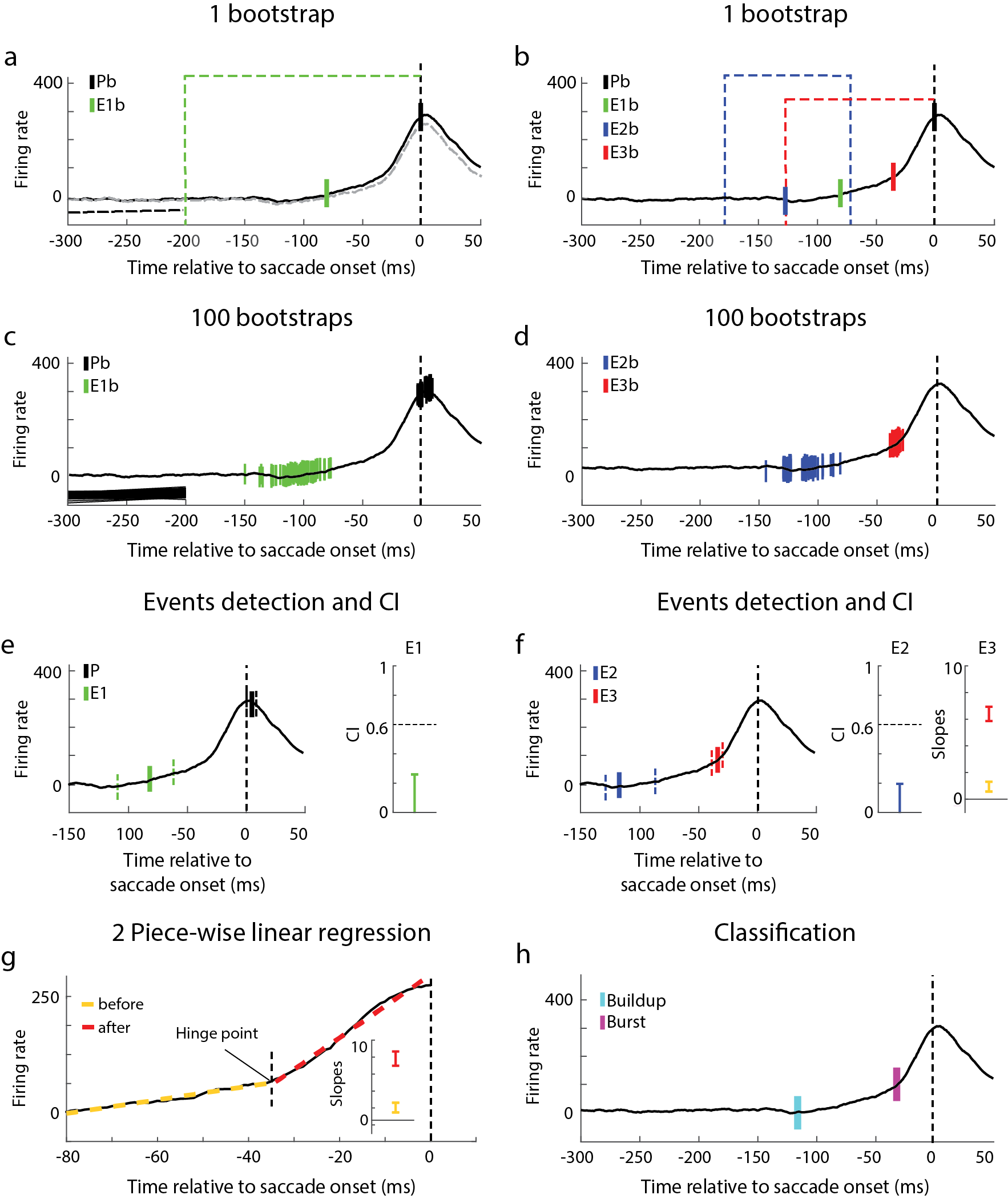


**Supplementary Figure 3. Events detection and classification of activity during the pre-saccadic epoch.** (a) Average spike density waveform for one channel is shown for one bootstrap iteration (solid black trace). The near-horizontal, dashed black line is the linear trend estimated in [-300 -200]ms window. This trend was extrapolated to the remaining time points and subtracted from the trial-averaged activity to yield the dashed gray waveform. The time and amplitude at peak activity (*Pb*) and when the detrended activity becomes significantly different from baseline (*E1b*) for this bootstrap iteration are shown in black and green tick marks, respectively. The green dashed region delimits the search window of this event. (b) The original spike density waveform (black traces) now also overlays the ‘hinge’ points denoting buildup (*E2b*; blue tick mark) and burst (*E3b*; red tick mark) onsets for one bootstrap iteration. The blue and red dashed lines mark the search windows of the two events. (c,d) The spike density waveform is now shown with the distribution of each event for 100 bootstrap iterations. Subplots are separated for visualization. (e) Left: The mean estimates and confidence intervals (CIs) of events *P* (black) and *E1* (green) obtained from the 100 bootstrap iterations are shown respectively as solid and dashed tick marks. They are superimposed on the trial-averaged spike density function. Right: The normalized range of CI for *E1* is shown relative to an arbitrarily chosen threshold level indicated by the horizontal dashed line. (f) Left: Same format is used to shown the mean and CIs for events *E2* (blue) and *E3* (red). Middle: The normalized range of CI for *E2* is shown relative the same threshold level indicated by the horizontal dashed line. Right: The plot shows the means and CIs of the slopes of the regression fits before (yellow) and after (red) the hinge point of event *E3*. (g) A visualization of a hinge point detection from a two-piece linear regression analysis. The dashed lines indicated the best fit lines before (yellow) and after (red) the hinge point. (h) The final step of the analysis is to classify events *E2* and *E3* into Buildup (cyan) and Burst (purple) events (see *Methods* for criterion details).

**Supplementary Figure 4**

**
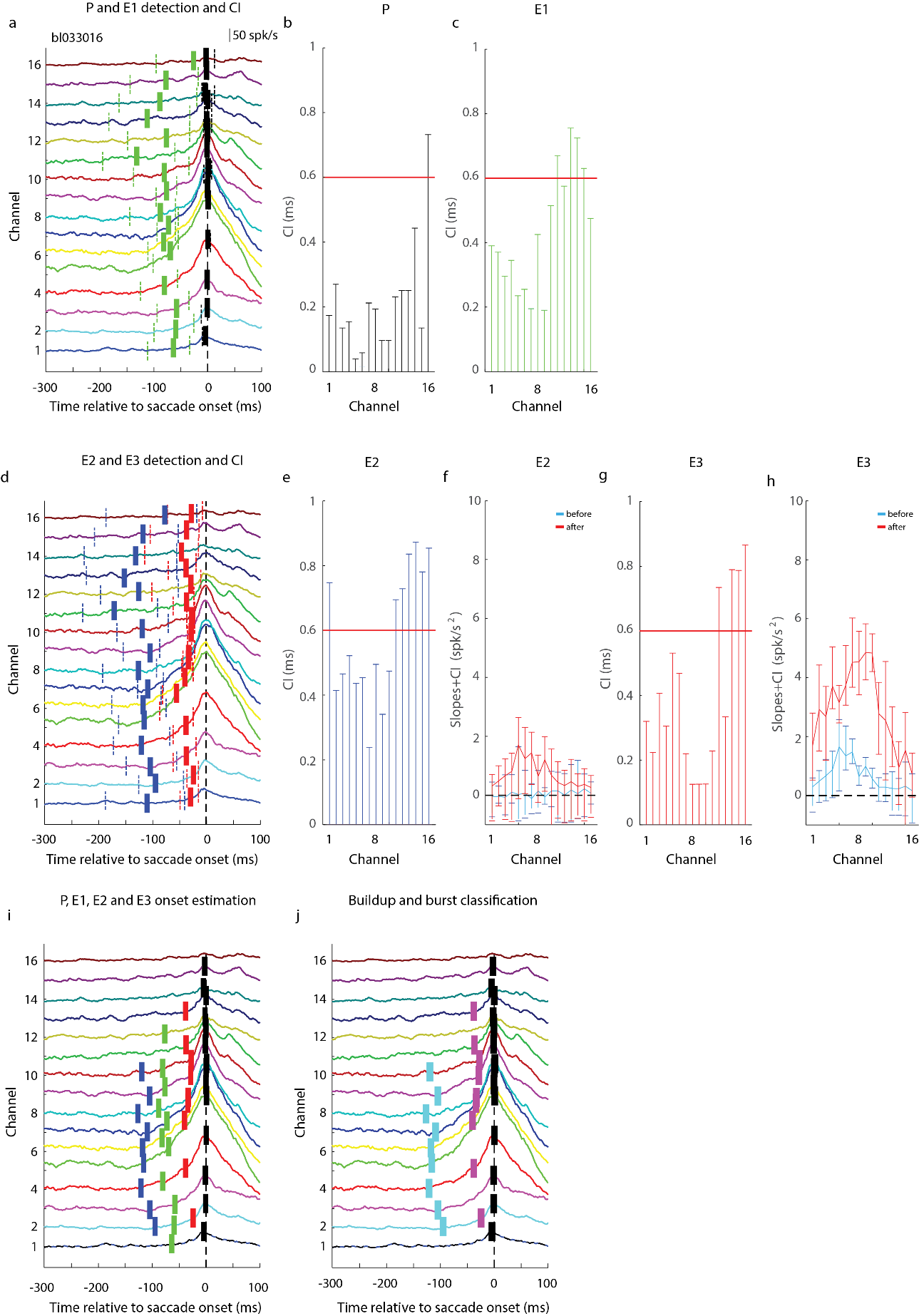
**

**Supplementary Figure 4. Example of events detection during the pre-saccadic epoch and classification.** The detection of all events and their CIs are illustrated for trial-averaged spike density functions on all channels for the VG task of an example session. Data are aligned on saccade onset. Mean event value is shown as a solid, thick vertical tick mark. CIs are denoted as dashed, thin vertical lines of the same color and on each side of the solid tick mark. Events *P*, *E1*, *E2* and *E3* are identified respectively in black, green, blue, and red colors. (a-c) Neural activity waveforms are shown with mean and CIs of events *P* and *E1*. (d-h) Neural activity waveforms are shown with mean and CIs of events *E2* and *E3*. (f,h) The mean and CIs of slopes of the linear regressions before (cyan) and after (red) the hinge points are plotted as a function of channel number for events *E2* and *E3*. (i) The spike density waveforms for all channels are now shown with average values of the statistically significant events (see *Methods* for details). (j) The events are now replaced classification into buildup (cyan) or burst (purple) events.

**Supplementary Figure 5.**

**
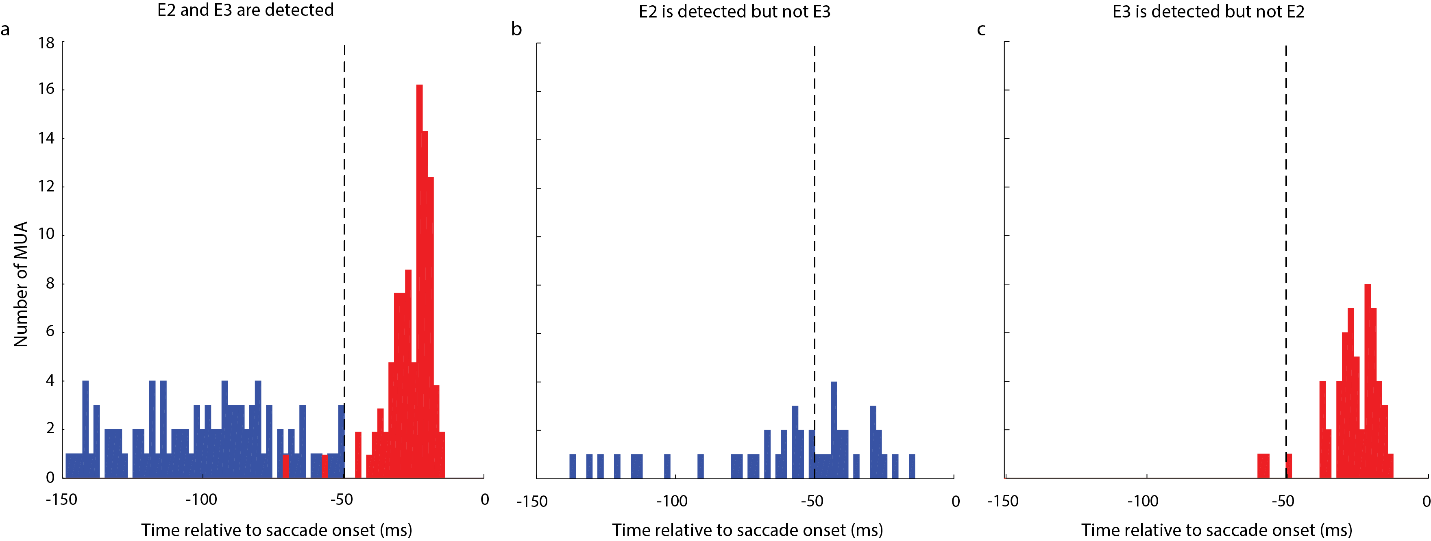
**

**Supplementary Figure 5. Example of events detection during the pre-saccadic epoch and classification: classification histograms.** (a-c) The events classification of Supplementary Figure 4i,j was determined from the distributions of events *E2* and *E3* and depending on whether only one or both events were detected. The vertical dashed line represents the -50ms boundary used to classify the detected events into Buildup and Burst activity (see *Methods*).

**Supplementary Figure 6**


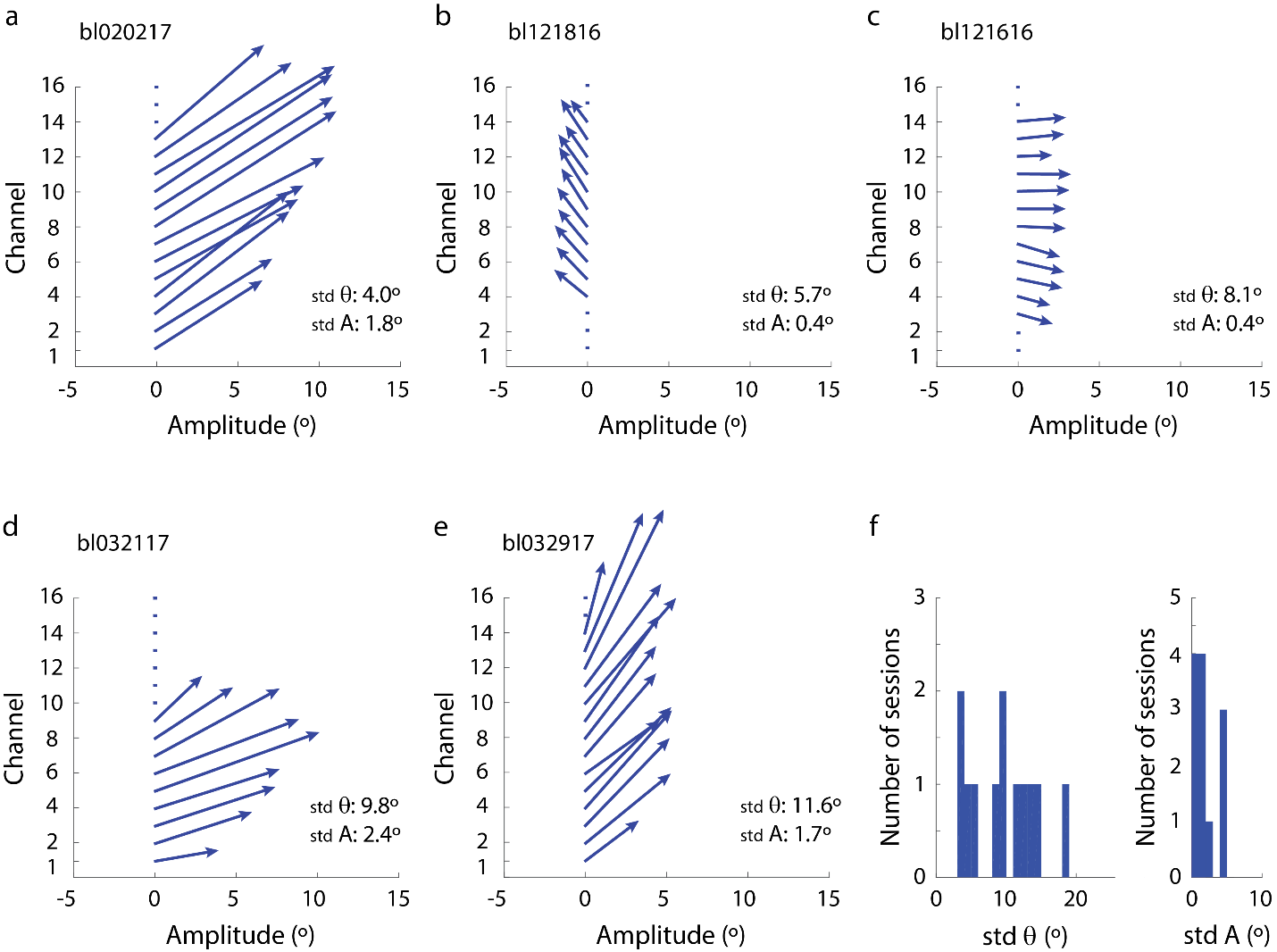


**Supplementary Figure 6.** Saccade vector distribution across depths and across sessions. (a-e) Vector of the saccadic eye movement induced by stimulation of each channel during example sessions; blue arrows indicate the direction and amplitude of the induced saccades; blue dots indicate the absence of saccades after the onset of the stimulation; bottom right corner displays the standard deviation of the direction ($\theta$) and amplitude (*A*) of the saccade vector for the example session. (f) Distribution of the standard deviation of the direction (left) and amplitude (right) across all sessions when the data from the stimulation paradigm was recorded (12 sessions).

**Supplementary Table 1**

|  | **VG** | | **MG** | |
| --- | --- | --- | --- | --- |
|  | # | % | # | % |
| **Total** | 254 | 100 | 149 | 100 |
| Visual-only | 16 | 6 | 14 | 9 |
| Visuo-movement | 180 | 71 | 113 | 76 |
| Movement-only | 58 | 23 | 22 | 15 |

**Supplementary Table 1. Categorization of SC neurons.** Summary of the percentages of each type of neuron for VG and MG trials.

**Supplementary Table 2**

|  | **VG** | | **MG** | |
| --- | --- | --- | --- | --- |
|  | # | % | # | % |
| **Total** | 203 | 100 | 149 | 100 |
| Buildup-only | 34 | 17 | 28 | 27 |
| Burst-only | 61 | 30 | 31 | 29 |
| Buildup-Burst | 108 | 53 | 47 | 44 |

**Supplementary Table 2. Classification of SC neurons based on pre-saccadic activity.** Summary of the percentages of each type of neuron for VG and MG trials.

**Supplementary Results and Discussion:**

Events *E1*, *E2, E3* and *P* detection and classification into Burst and Buildup

Supplementary Figure 4a-i shows the detection of events *E1*, *E2, E3* and *P* for the example dataset. The reliability of the detection of each event was measured by the CIs obtained through bootstrapping (see *Methods*). One can observe that for many channels CIs were well below the 0.6 threshold, indicating a reliable estimation of all events. Panels f and h also report the slopes and CIs around the hinge points for events *E2* and *E3*. The slopes were not significantly different for most of *E2* events, reflecting the slow accumulation of the activity. In contrast, they were significantly different for *E3*, reflecting a sharp increase of the activity. Supplementary Figure 4i shows the final estimation of all four events, provided that they were statistically significant (see *Neuronal activity categorization* in *Methods* section). Even though the search window for *E2* was large (within 100ms before *E1* and a total search window of 300ms), *E2* events were systematically detected within 50ms before E1, reflecting the onset of the accumulation that gives rise to *E1*.

The next step was to classify these events into Buildup or Burst activity. When both *E2* and *E3* were detected and were significantly different from each other (i.e., their CIs did not overlap), they were labeled as Buildup and Burst*s* events, respectively. If only *E2* was reliably estimated, the activity displayed an early Buildup (<-50ms) but without a reliable hinge point for *E3* (e.g., channel 3 of Supplementary Figure 4i). When only *E3* was reliably estimated, the activity only displayed a Burst just before saccade onset (>-50ms) but without an initial Buildup (e.g., channels 11 and 13 of Supplementary Figure 4i). We plotted the distributions of events *E2* and *E3* pooled across all channels and bootstrap iterations, although cases when both or only one of the two events were detected were considered separately (Supplementary Figure 5). It is important to note that the onset of each event is only constrained temporally by the size of their respective search windows, which means that either onset can occur at any time within 300ms before saccade onset with Buildup preceding Burst onset. The distributions of event times are binomial and exhibit visual separation around $-50ms$ relative to saccade onset. We therefore used this boundary to distinguish Buildup ($<-50ms$) from Burst ($>-50ms$) events. Thus, many events detected as *E2* (Buildup) were reclassified as *E3* (Burst). Supplementary Figure 4j replots the average spike density functions of all channels with identified Buildup and Burst events superimposed. For this session, peak activity (black tick marks) occurred around saccade onset for nearly all neurons along the dorsoventral dimension, Burst activity (purple tick marks) was also present in most neurons (but fewer than those with peak activity), while Buildup activity (cyan tick marks) was present primarily in neurons found along the ventral half of the track.

The analysis shown in Supplementary Figure 4 was performed for each laminar recording session. We then averaged across the sessions after depth aligning the data relative to the reference channel defined by the CSD method (see *Methods* and Supplementary Figure 1). Figure 7 summarizes the distribution of onset latencies and amplitudes of each event across depths and across sessions. A detailed description is given in the following sections.

Distributions of events *E1*, *E2*, *E3*, and *P* across sessions for VG task

Figure 7a show the results of the estimation of the events *E1*, *E2*, *E3*, and *P*. We found that peaks of activity were detected across the whole track (from channel -8 to 7, Figure 7 a,b (black trace)). Event *P* (black trace), which denotes the peak activity, was detected between -0.5ms (95%CI [-4.1 3.5]ms) on channel 7 and 5.9ms (95%CI [3.3 8.4]ms) on channel -2relative to saccade onset. Event *E1* (green trace), which corresponds to a significant change from baseline of the detrended activity during the 200ms preceding saccade onset, was detected between -76.0ms (95%CI [-101.0 -54.2]ms) and -33.8ms (95%CI [-44.6 -24.2]ms) relative to saccade onset. Event *E2* (blue trace), which detects the hinge point associated with the onset of the non-detrended activity accumulation in a 100ms window preceding *E1*, varied between -100.2ms (95%CI [-115.0 -84.6]ms) and -59.0ms (95%CI [-81.7 -35.8]ms) before saccade onset. Figure 7a highlights the tight relationship between *E1* and *E2*, with a maximum average difference of ~25ms and confidence intervals of the same order for both events, even though *E2* was searched for in a 100ms window preceding *E1*. We interpret this consistency to suggest that the ‘hinge point’ *E2* is a meaningful event. Event *E3* (red trace), identified as the ‘hinge point’ between *E2* and *P*, was detected between ­29.2ms (95%CI [-38.0 -23.1]ms) and -21.4ms (95%CI [-22.2 -20.5]ms) before saccade onset.

Figure 7b plots the amplitude of spiking activity at the time of event *P*, *E1*, *E2* and *E3* across depths. The activity at the onset of *E1* was limited to low firing rate, between 5.0spk/s(95%CI [2.2 9.4]spk/s) on channel -8 and 16.2spk/s(95%CI [4.0 37.1]spk/s) on channel 6. As expected, the average activity at the onset of *E2* was even closer to baseline than *E1*, and varied between -0.48spk/s (95%CI [-3.4 2.6]spk/s) and 7.8spk/s (95%CI [-3.2 25.5]spk/s). The activity at the onset of *E3* was variable across depths with a maximum amplitude of 54.4spk/s (95%CI [40.3 72.0]spk/s) on channel -4 and the minimum amplitude for the most dorsal and ventral channels. Finally, the activity at event *P* was similar to *E3* but with larger amplitude. Activity at *P* reached a maximum amplitude of 163.3spk/s (95%CI [134.6 192.7]spk/s) on channel -2 and a minimum amplitude for the most dorsal and ventral channels. Overall the results presented in Figure 7a,b indicate that events *E1*, *E2* and *E3* can be estimated reliably in the average waveform on each channel and that they correspond to systematic events of the pre-saccadic activity.

Distributions of events *E1*, *E2*, *E3*, and *P* across sessions for MG task

Figure 7e show the results of the estimation of the events *E1*, *E2*, *E3*, and *P*. We found that peaks of activity were detected across the whole track (from channel -8 to 7, Figure 7e,f (black trace)). Event *P* (black trace) was detected on average 1.3ms (95%CI [-2.0 4.8]ms) after saccade onset without any significant trend across channels (cubic fit, R^2^ =0.33, *P*=0.2 >0.05). Event *E1* (green trace) was detected between -65.1ms (95%CI [-88.1 -44.5]ms) and -30.2ms (95%CI [-52.4 -15.1]ms) relative to saccade onset. Event *E2* (blue trace) varied between -93.6ms (95%CI [-110.9 -76.3]ms) and -43.9ms (95%CI [-51.4 -36.5]ms) relative to saccade onset. Once again, Figure 7e highlights the tight relationship between *E1* and *E2*, with a maximum difference of ~30ms and confidence intervals of the same order for both events. Event *E3* (red trace) was detected on average -28.4ms (95%CI [-36.4 -22.1] ms) relative to saccade onset between channel -6 and 6, without any significant trend across depths (cubic fit, R^2^=0.10, *P*=0.80).

Figure 7f plots the amplitude of spiking activity at the time of event *P*, *E1*, *E2* and *E3* across depths. The activity at the onset of *E1* was limited to low firing rate between 3.6spk/s (95%CI [-4.7 10.7]spk/s) and 27.2spk/s (95%CI [15.6 37.7]spk/s). As expected, the average activity at the onset of *E2* was very close to baseline, between -1.1spk/s (95%CI [-10.6 9.2]spk/s) and 11.3spk/s (95%CI [4.4 18.0]spk/s). The activity at the onset of *E3* was variable across depths with a maximum amplitude of 63.8spk/s (95%CI [43.7 82.8]spk/s) on channel -2. The minimum amplitude for the most dorsal channel 26.9spk/s (95%CI [17.6 37.4]spk/s) on channel 4. Finally, the activity at event *P* reached a maximum amplitude of 132.8spk/s (95%CI [97.1 167.9]spk/s) on channel -1, which is ~30spk/s lower than the maximum amplitude for VG trials and a minimum activity for the most dorsal and ventral channels. Similar to VG trials, the results presented in Figure 7e,f indicate that events *E1*, *E2* and *E3* can be estimated reliably in the average waveform.

Stimulation-evoked saccades along the dorsoventral axis of SC

Although the focus of this study was to examine SC activity patterns across depth, we did occasionally deliver electrical stimulation through each contact at the end of experimental sessions. To preserve the integrity of the electrode, however, each contact was stimulated only a few times (typically just twice) and the same stimulation parameters (40µA, 400Hz, 200ms; biphasic, 200µs pulse duration, 17µs inter-pulse duration) were used across contacts and sessions. Sufficient data were available for 12 sessions to permit a preliminary evaluation. Supplementary Figure 6 plots stimulation-evoked saccade vectors as a function of depth for 5 example sessions. It also provides a histogram of the standard deviations in amplitude and direction across contacts for each session. All but one session had lower than 15^o^ of standard deviation in directions and all sessions had less than 5^o^ of standard deviation in amplitude. Hence, there was a high degree of similarity between saccade vectors across channels. Note that although the electrode was inserted roughly orthogonally to the surface of SC, these measurements are not sufficient to ensure that it traversed in an actual anatomical “column” within the SC.

Nomenclature of SC neurons.

The long history of SC studies of sensorimotor transformation, specifically visual input leading to saccadic eye movement, have yielded a variety of names to describe the types of neurons involved in the process. The most generic, hypothesis-free nomenclature is to classify them as visual, motor and visuomotor neurons based on activity modulation during the visual and/or movement intervals. Visual neurons are found in the superficial layers, and visuomotor and motor neurons reside in the deeper layers[^2^](#_ENREF_2)^,^[^3^](#_ENREF_3), but it has been debated whether the latter two are segregated along the dorsoventral axis. The ability of a laminar probe to record simultaneously the activities of neurons along this axis revealed that there is a gradual transition from visual to visuomotor to motor neurons with depth but the vast majority are visuomotor (Figure 4 and Supplementary Table 1). We also demonstrated previously[^4^](#_ENREF_4) and discussed above that putative motor neurons can exhibit a visual response under certain conditions. Thus, we prefer to avoid making a distinction between visuomotor and motor neurons.

Hypothesis-guided names have also been assigned to SC neurons, with different nomenclatures being introduced over time. Saccade-related burst neurons discharge a high-frequency burst for optimal vector saccades, and burst onset is tightly coupled to saccade onset, leading the movement by ~20ms[^2^](#_ENREF_2). Neurons with low-frequency activity several hundred milliseconds before the burst were once called long-lead movement neurons[^3^](#_ENREF_3)^,^[^5^](#_ENREF_5). They may be the same subclass of neurons that were later termed quasi-visual neurons [^6^](#_ENREF_6) and prelude neurons[^7^](#_ENREF_7). Other families of names (clipped, partially clipped, and unclipped neurons or open- and closed-movement field neurons) emerged from experiments that tested whether SC activity controls dynamic motor error[^8^](#_ENREF_8)^,^[^9^](#_ENREF_9). The current nomenclature labels intermediate/deep layer neurons as fixation, burst, and buildup neurons[^9^](#_ENREF_9)^,^[^10^](#_ENREF_10). Fixation neurons reside in the rostral pole of the SC. They discharge at a tonic rate during fixation, pause during large saccades, and burst during very-small amplitude saccades, including microsaccades[^10^](#_ENREF_10)^,^[^11^](#_ENREF_11). Buildup neurons exhibit low-level activity well before saccade onset and therefore are similar to prelude or long-lead movement neurons. Burst neurons are closest to the saccade-related burst neurons. Finally, the reader should keep in mind that despite the use of categories, most neurons exhibit both Burst and Buildup features (Figure 8 and Supplementary Table 2).

More recent SC studies have used a stochastic accumulator framework to correlate features of neural activity with saccade reaction time e.g.,[^12^](#_ENREF_12)^,^[^13^](#_ENREF_13)^,^[^14^](#_ENREF_14). Fitting the pre-saccadic activity with a two-piecewise linear regression yielded a time of inflection point that is correlated with reaction time[^13^](#_ENREF_13). Moreover, their data suggest that the accumulation occurs ~65ms before saccade onset (accumulation and saccade onsets were respectively 142$\pm$16ms and 207$\pm$20ms, relative to fixation offset). We sought to relate this finding to the Buildup or Burst features of neural activity, and an initial glance suggests that accumulation onset is a feature of Buildup neurons. However, our analysis suggests that Buildup onset occurs at least 30ms earlier, ~100ms before saccade onset (Figure 7). We believe this discrepancy may be a result of the differences in detection approaches. We developed a method to reliably detect and classify a Buildup (accumulation) and/or a Burst (threshold) process. By contrast, the previous study, by using a two-piece linear regression, limited the detection to only one neural event that spanned from 100 ms before fixation offset to the time of peak in the saccade-related burst[^13^](#_ENREF_13). Their method was applied on individual trials and the amount of stochastic noise in the spiking discharge may have prevented the distinction between two neural events. Here we used trial-averaged waveform combined with bootstrapping of the trial sets, which allowed the detection of at most two distinct neural events within a probabilistic framework. To the best of our knowledge, this is the first time a method is developed to detect precisely and reliably the onset of Buildup and Burst activity, beyond the use of predetermined temporal windows of analysis. This method revealed that Buildup activity was detected gradually on different channels spanning ~30ms since the initial Buildup around the center of the intermediate layers.

Burst onset occurred synchronously across all layers, ~28ms before saccade onset. This is comparable with the values used or estimated in previous oculomotor studies[^13^](#_ENREF_13)^,^[^15^](#_ENREF_15)^,^[^16^](#_ENREF_16). It is also in line with the spike modulation times observed in burst generator neurons that participate in saccade generation[^17^](#_ENREF_17)^,^[^18^](#_ENREF_18). It is generally stated the impact of reaching a threshold (equivalently, entering burst mode), either at individual or population activity, is to inhibit the brainstem omnipause neurons[^15^](#_ENREF_15)^,^[^19^](#_ENREF_19)^,^[^20^](#_ENREF_20), although other frameworks that depend on state space dynamical systems may facilitate direct communication between SC and pontine burst neurons[^21-23^](#_ENREF_21).

**REFERENCES**

1 Pettersen, K. H., Devor, A., Ulbert, I., Dale, A. M. & Einevoll, G. T. Current-source density estimation based on inversion of electrostatic forward solution: effects of finite extent of neuronal activity and conductivity discontinuities. *J Neurosci Methods* **154**, 116-133, doi:10.1016/j.jneumeth.2005.12.005 (2006).

2 Sparks, D. L. Functional properties of neurons in the monkey superior colliculus: coupling of neuronal activity and saccade onset. *Brain Res* **156**, 1-16 (1978).

3 Mohler, C. W. & Wurtz, R. H. Organization of monkey superior colliculus: intermediate layer cells discharging before eye movements. *J Neurophysiol* **39**, 722-744 (1976).

4 Jagadisan, U. K. & Gandhi, N. J. Disruption of Fixation Reveals Latent Sensorimotor Processes in the Superior Colliculus. *J Neurosci* **36**, 6129-6140, doi:10.1523/JNEUROSCI.3685-15.2016 (2016).

5 Wurtz, R. H. & Albano, J. E. Visual-motor function of the primate superior colliculus. *Annu Rev Neurosci* **3**, 189-226 (1980).

6 Mays, L. E. & Sparks, D. L. Dissociation of visual and saccade-related responses in superior colliculus neurons. *J Neurophysiol* **43**, 207-232 (1980).

7 Glimcher, P. W. & Sparks, D. L. Movement selection in advance of action in the superior colliculus. *Nature* **355**, 542-545, doi:10.1038/355542a0 (1992).

8 Waitzman, D. M., Ma, T. P., Optican, L. M. & Wurtz, R. H. Superior colliculus neurons mediate the dynamic characteristics of saccades. *J Neurophysiol* **66**, 1716-1737 (1991).

9 Munoz, D. P. & Wurtz, R. H. Saccade-related activity in monkey superior colliculus. I. Characteristics of burst and buildup cells. *J Neurophysiol* **73**, 2313-2333 (1995).

10 Munoz, D. P. & Wurtz, R. H. Fixation cells in monkey superior colliculus. I. Characteristics of cell discharge. *J Neurophysiol* **70**, 559-575 (1993).

11 Hafed, Z. M., Goffart, L. & Krauzlis, R. J. A neural mechanism for microsaccade generation in the primate superior colliculus. *Science* **323**, 940-943, doi:323/5916/940 [pii] 10.1126/science.1166112 (2009).

12 Reppert, T. R., Servant, M., Heitz, R. P. & Schall, J. D. Neural mechanisms of speed-accuracy tradeoff of visual search: saccade vigor, the origin of targeting errors, and comparison of the superior colliculus and frontal eye field. *J Neurophysiol* **120**, 372-384, doi:10.1152/jn.00887.2017 (2018).

13 Peel, T. R., Dash, S., Lomber, S. G. & Corneil, B. D. Frontal Eye Field Inactivation Diminishes Superior Colliculus Activity, But Delayed Saccadic Accumulation Governs Reaction Time Increases. *J Neurosci* **37**, 11715-11730, doi:10.1523/JNEUROSCI.2664-17.2017 (2017).

14 Ratcliff, R., Cherian, A. & Segraves, M. A comparison of macaque behavior and superior colliculus neuronal activity to predictions from models of two-choice decisions. *J Neurophysiol* **90**, 1392-1407 (2003).

15 Hanes, D. P. & Schall, J. D. Countermanding saccades in macaque. *Vis Neurosci* **12**, 929-937 (1995).

16 Jantz, J. J., Watanabe, M., Everling, S. & Munoz, D. P. Threshold mechanism for saccade initiation in the frontal eye field and superior colliculus. *J Neurophysiol* **109**, 2767-2780, doi:10.1152/jn.00611.2012 (2013).

17 Cullen, K. E. & Guitton, D. Analysis of primate IBN spike trains using system identification techniques. I. Relationship to eye movement dynamics during head-fixed saccades. *J Neurophysiol* **78**, 3259-3282 (1997).

18 Gandhi, N. J. & Keller, E. L. Activity of the brain stem omnipause neurons during saccades perturbed by stimulation of the primate superior colliculus. *J Neurophysiol* **82**, 3254-3267 (1999).

19 Keller, E. L. Participation of medial pontine reticular formation in eye movement generation in monkey. *J Neurophysiol* **37**, 316-332 (1974).

20 Zandbelt, B., Purcell, B. A., Palmeri, T. J., Logan, G. D. & Schall, J. D. Response times from ensembles of accumulators. *Proc Natl Acad Sci U S A* **111**, 2848-2853, doi:10.1073/pnas.1310577111 (2014).

21 Churchland, M. M., Yu, B. M., Ryu, S. I., Santhanam, G. & Shenoy, K. V. Neural variability in premotor cortex provides a signature of motor preparation. *J Neurosci* **26**, 3697-3712 (2006).

22 Kaufman, M. T., Churchland, M. M., Ryu, S. I. & Shenoy, K. V. Cortical activity in the null space: permitting preparation without movement. *Nat Neurosci* **17**, 440-448, doi:10.1038/nn.3643 (2014).

23 Jagadisan, U. K. & Gandhi, N. J. Population Temporal Structure Supplements The Rate Code During Sensorimotor Transformations. *bioRxiv*, 132514, doi:10.1101/132514 (2018).
