# Supplementary material for: Sensorimotor transformation elicits systematic patterns of activity along the dorsoventral extent of the superior colliculus in the macaque monkey
Source: Commun Biol. 2019 Aug 2;2:287. doi: 10.1038/s42003-019-0527-y (PMC6677725; doi:10.1038/s42003-019-0527-y)
Supplement: Supplementary file 2 — Reporting Summary [file 42003_2019_527_MOESM2_ESM.pdf]

nature research

Corresponding author(s): Corentin Massot
Neeraj J. Gandhi

Reporting Summary

Nature Research wishes to improve the reproducibility of the work that we publish. This form provides structure for consistency and transparency in reporting. For further information on Nature Research policies, see Authors & Referees and the Editorial Policy Checklist.

Statistical parameters

When statistical analyses are reported, confirm that the following items are present in the relevant location (e.g. figure legend, table legend, main text, or Methods section).

n/a	Confirmed	
☐	☒	The exact sample size (*n*) for each experimental group/condition, given as a discrete number and unit of measurement
☐	☒	An indication of whether measurements were taken from distinct samples or whether the same sample was measured repeatedly
☐	☒	The statistical test(s) used AND whether they are one- or two-sided<br>*Only common tests should be described solely by name; describe more complex techniques in the Methods section.*
☒	☐	A description of all covariates tested
☐	☒	A description of any assumptions or corrections, such as tests of normality and adjustment for multiple comparisons
☐	☒	A full description of the statistics including central tendency (e.g. means) or other basic estimates (e.g. regression coefficient) AND variation (e.g. standard deviation) or associated estimates of uncertainty (e.g. confidence intervals)
☐	☒	For null hypothesis testing, the test statistic (e.g. *F*, *t*, *r*) with confidence intervals, effect sizes, degrees of freedom and *P* value noted<br>*Give P values as exact values whenever suitable.*
☒	☐	For Bayesian analysis, information on the choice of priors and Markov chain Monte Carlo settings
☒	☐	For hierarchical and complex designs, identification of the appropriate level for tests and full reporting of outcomes
☒	☐	Estimates of effect sizes (e.g. Cohen's *d*, Pearson's *r*), indicating how they were calculated
☐	☒	Clearly defined error bars<br>*State explicitly what error bars represent (e.g. SD, SE, CI)*

Our web collection on statistics for biologists may be useful.

Software and code

Policy information about availability of computer code

Data collection	Neurophysiological data: Ripple system (multi-channel scout system, Salt Lake City, USA). Oculomotor data: EyeLink 1000 (SR Research, Ottawa, Canada). Overall behavior and neural data acquisition: real-time in-house system (Bryant and Gandhi, 2005). Custom MATLAB scripts (Mathworks, Natick, USA)
Data analysis	Custom MATLAB scripts (Mathworks, Natick, USA)

For manuscripts utilizing custom algorithms or software that are central to the research but not yet described in published literature, software must be made available to editors/reviewers upon request. We strongly encourage code deposition in a community repository (e.g. GitHub). See the Nature Research guidelines for submitting code & software for further information.

Data

Policy information about availability of data

All manuscripts must include a data availability statement. This statement should provide the following information, where applicable:

- Accession codes, unique identifiers, or web links for publicly available datasets
- A list of figures that have associated raw data
- A description of any restrictions on data availability

All data are available upon request to the corresponding authors for fair use.

Field-specific reporting

Please select the best fit for your research. If you are not sure, read the appropriate sections before making your selection.

☒ Life sciences ☐ Behavioural & social sciences ☐ Ecological, evolutionary & environmental sciences

For a reference copy of the document with all sections, see nature.com/authors/policies/ReportingSummary-flat.pdf

Life sciences study design

All studies must disclose on these points even when the disclosure is negative.

Sample size	The sample size was determined by the maximum number of penetrations of the laminar probe in the superior colliculus allowing the recording of neural activity with sufficient signal-to-noise ratio.
Data exclusions	6 sessions were discarded due to poor signal-to-noise ratio of the spiking activity and/or noisy LFP signals.
Replication	Replication was verified by recording neural activity during the exact same tasks in two different animals.
Randomization	Each population of neurons was associated to the animal it was recorded from. Randomization was not relevant to the study. Neural activity was recorded in the same experimental conditions for the two animals and for all sessions. The inclusion of covariates was not relevant to the study.
Blinding	Blinding was not relevant to the study as data analyses required the identification of the animal to verify the replication of the obtained results.

Reporting for specific materials, systems and methods

Materials & experimental systems

n/a	Involved in the study	
☒	☐	Unique biological materials
☒	☐	Antibodies
☒	☐	Eukaryotic cell lines
☒	☐	Palaeontology
☐	☒	Animals and other organisms
☒	☐	Human research participants

Methods

n/a	Involved in the study	
☒	☐	ChIP-seq
☒	☐	Flow cytometry
☒	☐	MRI-based neuroimaging

Animals and other organisms

Policy information about studies involving animals; ARRIVE guidelines recommended for reporting animal research

Laboratory animals	Two males, rhesus macaque monkeys (Macaca mulatta, ages 13 and 8 years).
Wild animals	The study did not involve wild animals
Field-collected samples	The study did not involve samples collected from the field.
